# Shifting Perspectives on the Role of Tocotrienol vs. Tocopherol in Brain Health: A Scoping Review

**DOI:** 10.3390/ijms26136339

**Published:** 2025-06-30

**Authors:** Rabiatul Adawiyah Razali, Wan Zurinah Wan Ngah, Suzana Makpol, Daijiro Yanagisawa, Tomoko Kato, Ikuo Tooyama

**Affiliations:** 1 Medical Innovation Research Center (MIRC), Shiga University of Medical Science, Otsu 520-2192, Shiga, Japan; rabiatul@belle.shiga-med.ac.jp (R.A.R.); tkato@belle.shiga-med.ac.jp (T.K.); kinchan@belle.shiga-med.ac.jp (I.T.); 2 Department of Biochemistry, Faculty of Medicine, Universiti Kebangsaan Malaysia, Cheras, Kuala Lumpur 56000, Malaysia; suzanamakpol@ukm.edu.my; 3 Molecular Neuroscience Research Center (MNRC), Shiga University of Medical Science, Otsu 520-2192, Shiga, Japan; daijiroy@belle.shiga-med.ac.jp

**Keywords:** vitamin E isomers, cognitive function, neuroprotection, dietary supplementation

## Abstract

Vitamin E has been extensively studied for its neuroprotective properties, with increasing evidence supporting its broader roles in brain health. This scoping review aims to systematically identify, analyze, and synthesize evidence of the existing literature over the last 10 years on tocotrienol and tocopherol supplementation in humans. A systematic search was conducted across PubMed, Scopus, and EBSCOhost yielding 42 eligible articles. Findings suggest that tocopherols, especially α- and γ-forms, are associated with improved cognitive performance, reduced neuroinflammation, and preservation of synaptic proteins. Despite tocotrienol’s lower plasma bioavailability, tocotrienol availability in selective brain regions has been associated with structural protection, particularly in white matter. Both compounds exhibit complementary effects, suggesting a potential advantage of combined supplementation. However, heterogeneity in study designs, subject characteristics, dosage, duration, and assessment methods limit direct comparisons and generalizability of findings. Based on our review’s findings, further research such as dose-optimization, long-term exposures, and delivery methods on human studies should be performed. This review highlights the multifaceted roles of vitamin E in brain health and underscores the urgent need for well-designed studies to clarify the distinct and synergistic effects of tocopherols and tocotrienols, particularly in human populations.

## 1. Introduction

Vitamin E refers to a group of fat-soluble compounds with potent antioxidant properties that contribute to various physiological functions, including immune regulation, cardiovascular protection, and cellular defense mechanisms [1,2,3,4]. They play a central role in protecting cellular structures from oxidative stress. Its antioxidant function involves scavenging reactive oxygen species (ROS) and reactive nitrogen species (RNS), thereby preventing lipid peroxidation in cell membranes, lipoproteins, and mitochondrial structures [5]. This protective role is important for tissues with high oxidative activity, such as the brain, liver, and cardiovascular system. In addition to its direct antioxidant effects, vitamin E also modulates intracellular signaling pathways that influence inflammatory responses [6]. Through these combined mechanisms, vitamin E not only defends against oxidative insults but also actively contributes to fighting inflammation, making it an important player in redox balance and immune homeostasis across multiple tissues.

Due to its potent properties, research on Vitamin E’s role in supporting brain health has garnered vast attention. Structurally, the brain is highly susceptible to oxidative stress due to its high lipids’ composition and elevated oxygen composition. These factors make it especially susceptible to the harmful effects of reactive oxygen species (ROS), which potentially contribute to neurodegeneration [7,8]. The capability of vitamin E to scavenge free radicals, regulate inflammatory pathways, and maintain membrane stability makes it a significant contributor to brain health [9,10,11,12].

**Figure 1:**
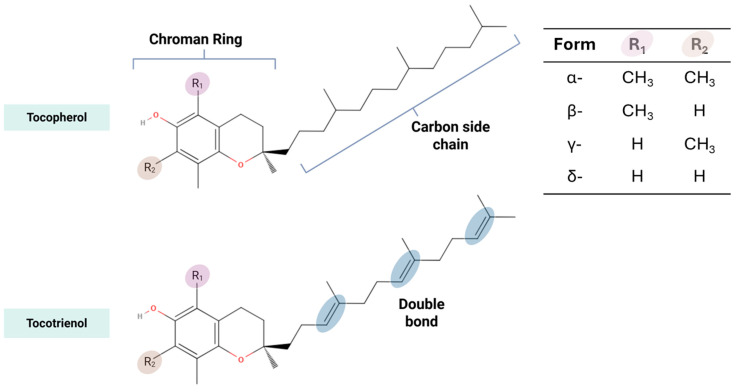
Structural comparison of Tocopherol and Tocotrienol (Vitamin E forms). “R” represents the variable side chains of vitamin E isomers.

Vitamin E is found both in natural sources and as a chemically synthesized compound. Naturally, it is abundant in cod liver oil, meat, milk, eggs and plant-based foods, particularly vegetable oils, grain, nuts, seeds, and leafy green vegetables [13,14]. Structurally, vitamin E is composed of tocopherol and tocotrienol, with each existing in four isomeric forms (α, β, γ, and δ) (Figure 1). These isomers differ in the number and position of methyl groups attached to the chromanol ring, which affects their biological activity, antioxidant capacity, and tissue distribution (Figure 1) [15].

Tocopherol’s structure consists of a saturated phytyl tail and it is found in a variety of food sources such as vegetable oils, nuts, and seeds [16,17]. Among the four isomers, α-tocopherol is typically regarded as the main functional form due to its efficient retention and antioxidant activity [7,18]. In addition to the naturally occurring forms, α -tocopherol has also been synthetically produced. The synthetic form of α-tocopherol, known as all-rac-α-tocopherol, consists of a mixture of eight stereoisomers resulting from three chiral centers at positions 2, 4′, and 8′. These include RRR, RSR, RRS, RSS, SRR, SRS, SSR, and SSS. In the all-rac-α-tocopherol, RRR-α-tocopherol only makes up 12.5% in the mix and is the only naturally occurring form in it [19,20].

Approximately 90% of vitamin E detected in both plasma and brain tissue samples is α-tocopherol due to the high affinity of vitamin E transport proteins for α-tocopherol [7]. Upon digestion in the stomach and duodenum, Vitamin E enters the enterocytes in the jejunum and is incorporated into chylomicrons (fat transport molecules) [21]. These molecules will carry the entire family of vitamin E through the lymph and the blood vessels. However, once these compounds reach the liver, α-tocopherol is preferentially recognized by α-tocopherol transfer protein (α-TTP), which actively incorporates it into very low-density lipoproteins (VLDLs) for secretion back into the bloodstream, delivering it to tissues or storage sites. The other forms of vitamin E are not efficiently retained, metabolized and excreted [22,23]. The binding of other forms of vitamin E, such as γ-tocopherol and tocotrienol, to α-TTP is less effective, resulting in correspondingly lower plasma concentrations.

In addition, the non-α-tocopherol compounds that are not preferentially bound by α-TTP will undergo metabolism through the hydroxylation pathway by enzymes such as the CYP450 superfamily [24]. Their metabolites are water-soluble and are excreted in the urine. Although other forms of tocopherol and tocotrienols are present in lower concentrations in circulation, studies have shown their beneficial effects. A recent comprehensive review on γ-tocopherol has shown that, this vitamin E isomer exerts beneficial health effects in humans by their antioxidant, anti-inflammatory, and anticancer properties [25]. γ-tocopherol effectively neutralizes reactive oxygen species (ROS) and reactive nitrogen species (RNS) and mediates anti-inflammatory effects through modulation of pathways involving cyclooxygenase-2 (COX-2) and tumor necrosis factor-α (TNF-α), hence providing cellular protection and reducing inflammation [25]. Although β- and δ-tocopherols are less studied, they were also shown to contribute to antioxidant defense mechanisms [16]

Tocotrienols, another class of vitamin E, found in dietary sources such as palm oil and rice bran, have been studied for their ability to reduce cholesterol levels, modulate cellular signaling pathways, and provide neuroprotective benefits in neurodegeneration [26,27]. Tocotrienol possesses an unsaturated isoprenoid side chain, which enhances their mobility in cell membranes and potentially allows better distribution in specific tissues such as the brain, skin, and liver [28,29,30]. Despite having a binding affinity to α-TTP of only 12% that of α-tocopherol [31], oral supplementation of tocotrienol was shown to increase in various types of tissue including blood, skin, adipose, brain, cardiac muscle and liver. [32]. Tocotrienols possess various beneficial properties such as a potent antioxidant and anticancer properties, anti-inflammatory effects, and cholesterol-lowering activity [33,34,35,36]. They also play a role in modulating gut microbiome balance [37] and, importantly, exhibit neuroprotective potential, particularly in the context of neurodegenerative diseases [3,38].

Despite evidence of vitamin E’s beneficial effect on brain health, there is still much debate on how effectively tocopherols versus tocotrienols function in brain wellness. While α-tocopherol has been emphasized in dietary recommendations, recent studies suggest that tocotrienols may provide complementary or even superior neuroprotective effects, especially in reducing neuroinflammation and oxidative damage [39,40]. The interaction of various vitamin E isomers and their overall effect on brain function remain an interesting area for investigation.

This scoping review aims to provide a comprehensive overview of the role of tocopherols and tocotrienols in brain health and their potential as dietary supplements to enhance cognitive performance. By examining the existing literature on their effects on cognitive performance, neuroinflammation, oxidative stress, brain structure, and neuroprotection, this scoping review will provide insights into the mechanisms through which vitamin E supports cognitive longevity and highlights key areas for future research.

## 2. Methods

### 2.1. PICO Framework and Research Question

This scoping review was guided by the PICO framework, which helped structure the research question and guide the evidence charting process [41]. PICO refers to Population (P), Intervention (I), Comparison (C), and Outcome (O). In this review, the population includes human subjects of various age groups. The intervention consists of the intake or circulating levels of tocotrienol and/or tocopherol. The comparison includes studies assessing these compounds against a placebo, no treatment, or baseline conditions, as well as studies directly comparing the two forms of vitamin E. The outcomes of interest focus on brain health, including cognitive function, neuroprotection, and the progression of neurodegeneration. Aligned with the PICO framework, the following research questions were developed to guide the scope and synthesis of this review: (1) What are the effects of tocotrienol or tocopherol supplementation on brain-health indicators? (2) How does tocotrienol differ from tocopherol in the maintenance of brain health?

### 2.2. Protocol

The protocol used for this scoping review was developed using the Preferred Reporting Items for Systematic Reviews and Meta-Analyses extension for Scoping Reviews (PRISMA-ScR) [42].

### 2.3. Eligibility Criteria

Article selection was performed according to the eligibility criteria. The criteria of articles to be included were (1) written in English; (2) published within a 10 year period; (3) human health study: randomized controlled trials (RCTs), observational studies, cohort studies, case–control studies, or cross-sectional studies; (4) investigating tocotrienol, tocopherol, or both (as supplements or dietary sources) independently or together; and (5) reporting on brain health-related outcomes. Studies that were (1) in the form of review articles, notes, letters, book chapters, or commentaries without original data; (2) published before 2014; (3) not published in English; (4) not investigating tocotrienol or tocopherol level or supplementation; and (5) not reporting on brain-health-related outcomes were excluded from this review.

### 2.4. Sources Database, Search Strategy and Selection of Evidence

A comprehensive search of the relevant words or phrases was performed on 6th January 2025 in PubMed (National Centre for Biotechnology Information, NCBI, Bethesda, MD, USA), Scopus (Elsevier, Amsterdam, The Netherlands), and EBSCOhost (EBSCO, Ipswich, MA, USA). The search strategies used the following words or phrases: (“Feedback, Sensory” [Mesh]) OR (“Cognition” [Mesh]) OR (“Memory” [Mesh]) OR (“Attention” [Mesh]) OR (“Brain” [Mesh]) OR (“Neuroprotection” [Mesh]) OR (“Aging” [Mesh]) OR (“Neuroimmunomodulation” [Mesh]) AND (“Tocopherols” [Mesh]) OR (alpha tocopherol) OR (beta tocopherol) OR (gamma tocopherol) OR (delta tocopherol) OR (“Tocotrienols” [Mesh]) OR (alpha tocotrienol) OR (beta tocotrienol) OR (gamma tocotrienol) OR (delta tocotrienol). Duplicate findings were sorted out first using EndNote 20.6 and further sorted out manually by the author. The articles were screened by examining the title and abstract based on the inclusion and exclusion criteria. Subsequently, a manual search was conducted by reviewing the reference lists of all included studies using a snowballing approach. Titles and abstracts of cited articles were screened against the same predefined inclusion and exclusion criteria to identify additional eligible human studies related to tocopherol, tocotrienol, and brain health.

### 2.5. Data Extraction, Charting and Data Item

A data-charting form was developed to determine which variables to extract. From the selected articles, data were extracted from texts, tables, figures, and supplementary materials in references and input into the data-charting form. The initial findings were arranged in a data extraction table, which includes the following: (1) source; (2) study design; (3) participant age group; (4) vitamin E form examined (5) vitamin E assessment method; (6) primary study focus; (7) brain-health outcome; and (8) brain-health theme.

### 2.6. Synthesis of Results

To answer the review question of what are the effects of tocotrienol or tocopherol supplementation on brain-health indicators, the obtained data were grouped according to brain-health indicators: (1) neuroinflammation and oxidative stress, (2) brain structure and function, (3) cognitive performance, and (4) neuroprotection and neurodegenerative disease alongside their outcome. The data was tabulated accordingly. For (1) neuroinflammation and oxidative stress and (2) brain structure and function, evaluated markers or brain region affected, together with their outcomes, were tabulated, and the outcome measures were visualized according to the value, using arrows (↓ = lower concentration, ↑ = higher concentration). For (3) cognitive performance, the effect direction for the outcome domain was used [43]. The outcome for each measure was visualized by using the upward, downward, or bidirectional arrow (upward arrow ▲ = positive score, downward arrow ▼ = negative score, sideways arrow ◀▶ = no change/mixed effects/conflicting findings). For (4) neuroprotection and neurodegenerative disease, the effect of vitamin E on diseases were listed accordingly.

## 3. Results

### 3.1. Sources of Evidence

**Figure 2:**
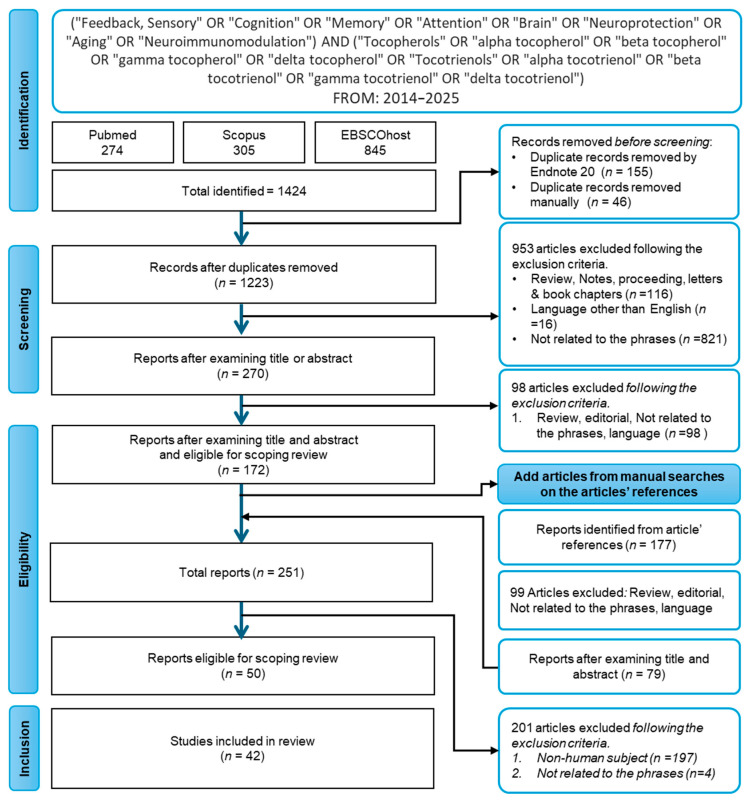
The PRISMA flow diagram illustrates the identification, screening, eligibility, and inclusion process of studies related to tocopherols and tocotrienols and their effects on brain health from 2014 to 2025. A total of 1424 articles were retrieved from PubMed, Scopus, and EBSCOhost databases using MeSH terms and keywords related to brain health and vitamin E isomers. Finally, 42 studies are selected for the review. Blue arrows indicate the flow of records through each stage from identification, screening, and eligibility, to final inclusion. Black arrows represent the filtering process at each stage, including exclusions based on predefined criteria and additions from manual reference searches.

A total of 1424 articles were obtained using PubMed (*n* = 274), Scopus (*n* = 305), and EBSCOhost (*n* = 845) (Figure 2). After sorting duplicates (*n* = 201), 1223 unique records remained for screening. Initially, 953 articles were excluded due to being review articles, non-English language, and irrelevant to the study’s terms. Upon further screening, 98 more were disqualified due to not fulfilling the inclusion criteria. From the 172 included articles, manual screening of the reference list yielded 177 new unique articles, of which 99 were disqualified because they were reviews, editorials, or irrelevant material. A total of 79 articles from the reference were added to the 172 initially included articles, resulting in a total of 251 articles eligible for review. However, due to the inclusion of human subjects only, 201 articles were excluded. Ultimately, 42 studies were included out of 50 reports that qualified for this scoping review.

### 3.2. General Characteristics of the Included Studies

**Figure 3:**
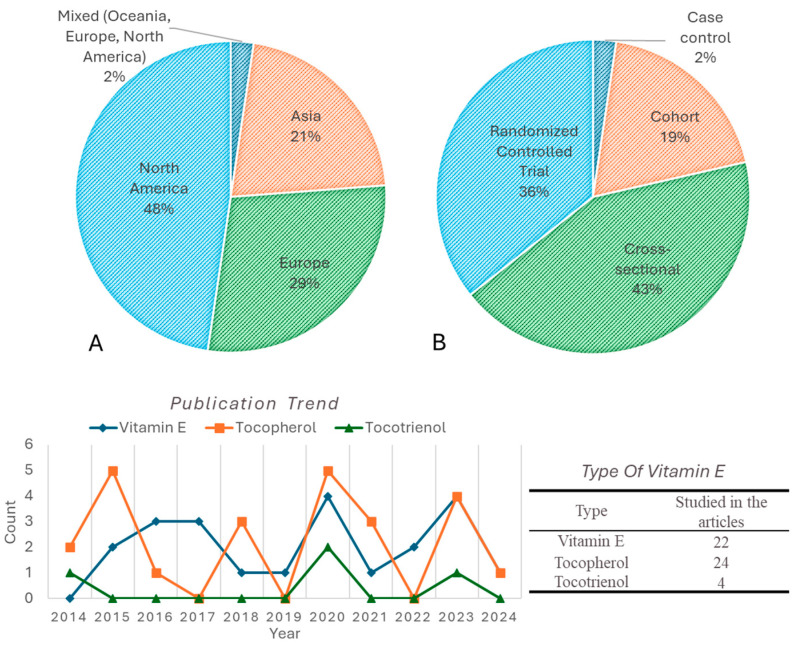
Overview of the general characteristics of the included studies. (**A**) Geographic distribution of studies included in the review, showing the proportion conducted in North America, Europe, Asia, and mixed regions. (**B**) Distribution of study designs, including cross-sectional, randomized controlled trials, cohort, and case–control studies. The graph shows the publication trend of vitamin E, tocopherol and tocotrienol for the past 10 years. The accompanying table summarizes the frequency of different vitamin E forms investigated across all included studies, highlighting tocopherol as the most frequently studied compound.

The geographical distribution indicates that most studies were conducted in North America (48%), followed by Europe (29%) and Asia (21%), with a small proportion in mixed regions (Figure 3A). The study design distribution showed that the majority were cross-sectional studies (43%), followed by randomized controlled trials (36%), cohort studies (19%), and case–control studies (2%) (Figure 3B). Overall, based on the trend of publications for the past decade, investigations of vitamin E effects on brain health have remained consistent but limited. Tocopherol studies were the highest number of publications overall with a notable spike occurring in 2015, 2018 and 2020. α-Tocopherol is the most investigated study (22 studies), followed by γ-tocopherol (17 studies). However, tocotrienol has been understudied and consistently has had a lower publication count over the past decade.

### 3.3. Overview of Vitamin E and Brain-Health Research for the Past 10 Years

All 42 articles eligible for this review were tabulated in Table 1. These articles examine the relationship between vitamin E and brain health across various populations and study designs. Many of these studies employed a cross-sectional approach, assessing various forms of vitamin E, including α-tocopherol, γ-tocopherol, and tocotrienols, through surveys, blood tests, or brain extractions. The research primarily focuses on cognitive performance, Alzheimer’s disease, Parkinson’s disease, dementia, and neurodegenerative markers such as amyloid plaques and neurofibrillary tangles. Methods of assessment vary widely, ranging from cognitive tests such as the Mini-Mental State Examination (MMSE) and the Consortium to Establish a Registry for Alzheimer’s Disease (CERAD) test to conduct genetic analyses of APOE genotypes. Additionally, several cohorts and randomized controlled trials have shown the potential neuroprotective effects of vitamin E by measuring oxidative stress, inflammatory cytokines, and biomarkers of neurodegeneration. Most of the studies involved elderly participants (60+ years), though some included younger adults and special populations such as schizophrenia or epilepsy patients. Due to variations in study design, study focus and outcome, the extracted studies were divided into four main themes related to brain health: neuroinflammation and oxidative stress, brain structure and function, cognitive performance, and neuroprotection and neurodegenerative diseases.

### 3.4. Neuroinflammation and Oxidative Stress

Neuroinflammatory and oxidative stress are among the brain-health themes that have been recognized in the reviewed articles. Markers of neuroinflammation and oxidative stress identified included inflammatory cytokines (e.g., GM-CSF, IL-8, IL-17, INF-α2), exosomal miRNAs (e.g., miR-122, miR-21), microglial activation, oxidative and nitrosative stress ratios (α-tocopherylquinone/α-tocopherol, 5-nitro-γ-tocopherol/γ-tocopherol), lipid peroxidation products (malondialdehyde, nitrotyrosine), homocysteine (tHcy), total antioxidant status (TAS), antioxidant enzymes (catalase, glutathione, SOD), serum tocopherol levels, leukocyte telomere length (LTL), and carotid intima-media thickness (C-IMT) (Table 2).

In Alzheimer’s disease (AD) patients, decreased α-tocopherol levels were associated with elevated inflammatory cytokines (G-CSF, GM-CSF, INF-α2, IL-3, IL-17, and IL-8) and reduced levels of several exosomal microRNAs (miR-122, miR-21, miR-132) [13]. In contrast, another study has shown that higher α- and γ-tocopherol levels in the brain were associated with reduced microglial activation, which shows vitamin E’s anti-inflammatory potential [1].

Additionally, vitamin E also have a protective effect towards oxidative stress. In the patients of AD with lower tocopherol and tocotrienol levels, oxidative (α-tocopherylquinone/α-tocopherol) and nitrosative (5-nitro-γ-tocopherol/γ-tocopherol) stress ratios were found to be increased [55]. Furthermore, multiple studies included in this review also reported that vitamin E supplementation can reduce lipid peroxidation (malondialdehyde, nitrotyrosine) and enhance antioxidant capacity, catalase, glutathione, and SOD levels in humans [4,59,66].

However, in individuals at ultra-high risk of psychosis who received omega-3 PUFA supplementation, blood analysis revealed a decrease in total glutathione (GSHt) levels, despite an increase in alpha-tocopherol (vitamin E) concentration [63]. Another study showed that centenarians with higher brain and serum levels of α-tocopherol, lutein, and β-carotene performed better cognitively. In contrast, centenarians with a higher n-6/n-3 PUFA ratio exhibited poorer cognitive performance [57].

In relation to neuroinflammation and oxidative stress, vascular and cellular aging were also associated with the level of vitamin E. Low α-tocopherol was linked to increased carotid intima-media thickness [67], while higher γ-tocopherol and total vitamin E levels correlated with longer leukocyte telomere length [55], suggesting anti-aging effects. In another study related to cognitively impaired patients, homocysteine (tHcy) was elevated; however, there is no direct link to vitamin E [51].

### 3.5. Brain Structure and Function

Besides its ability to modulate neuroinflammation and oxidative stress in the brain, vitamin E, particularly α- and γ-tocopherols, has been shown to have region-specific effects on brain structure and function. Approximately 12 studies from the last decade have discussed the physical effects of vitamin E on the brain (Table 3). The brain regions studied include the frontal cortex, midfrontal cortex, inferior frontal gyrus, hippocampus, subiculum, entorhinal cortex, amygdala, temporal cortex, inferior temporal cortex, visual cortex, parietal cortex, putamen, caudate, middle and superior frontal gyri, middle temporal gyrus, white matter, grey matter, and subcortical regions.

In the frontal cortex of the infant brain, concentrations of natural RRR-α-tocopherol were significantly higher than those of synthetic stereoisomers, providing early evidence that the brain selectively prefers certain specific forms of vitamin E [20]. In the same region, in the midfrontal cortex of elderly subjects, higher γ-tocopherol levels were positively associated with increased levels of several presynaptic proteins essential for synaptic function and cognitive health, while α-tocopherol showed no such association [7].

The hippocampus, which plays a role in memory and learning, also shows a strong preference for the natural RRR α-tocopherol and exhibits an inverse relationship between α-tocopherol levels and NFT counts in early stages of Alzheimer’s disease [12,20]. This protective effect of α-tocopherol was also observed in other early-affected regions, i.e., entorhinal cortex, amygdala, and subiculum [12]. Interestingly, no significant associations were observed in later-affected neocortical regions such as the temporal, frontal, and parietal cortices [12]. Additionally, the visual cortex exhibited the lowest α-tocopherol levels, indicating a lower demand for tocopherol in this region [20]. On the other hand, another form of tocopherol, γ-tocopherol, was found to be significantly associated with lower Alzheimer’s pathology across multiple brain regions, including the hippocampus, prefrontal cortex, temporal cortex, putamen, and caudate [9]. Total brain weight did not differ significantly by Alzheimer’s pathology severity [10]. However, it has been shown that tocopherol levels were significantly lower in both grey and white matter in Alzheimer’s patients, with a greater reduction in white matter [48].

Various effects of vitamin E on specific brain regions have also been observed in the reviewed studies, particularly in areas such as the frontal cortex, temporal cortex, hippocampus, and amygdala. Cortical regions had higher α-tocopherol concentrations and lower microglial activation compared to the subcortical region where microglial activation was higher [1]. In the inferior frontal gyrus, vitamin E’s effects on brain volume are influenced by haptoglobin (Hp) genotype, with Hp 2-1 carriers showing increased volume and Hp 1-1 carriers showing volume loss [54]. In addition, tocotrienol intake reduced white matter lesion progression [3]. Supplementation with vitamin E is also able to reduce seizure frequency and improve EEG [4].

### 3.6. Cognitive Performance

Over the past decade, cognitive performance has consistently been the primary focus of research on the effect of vitamin E on human brain health. Based on the articles reviewed, there are two primary approaches in examining the effects of vitamin E on cognition which are (1) assessing vitamin E levels in the body, and (2) conducting intervention or dietary intake studies. The summary of the findings is tabulated using the effect direction for the outcome domain.

#### 3.6.1. Level of Vitamin E

In this approach, researchers assessed vitamin E concentrations through blood or brain tissue analysis, or via nutritional surveys, at a single point in time. Overall, findings suggest that vitamin E, especially α-tocopherol and γ-tocopherol, showed variable associations with cognitive outcomes depending on factors such as study design, population and biomarker content.

Several studies reported positive associations between higher α-tocopherol levels and cognitive outcomes. Higher level of α-tocopherol was associated with better cognitive ability, improved daily living skills, and enhanced executive function [13,51,57]. Similarly, Alghadir et al. observed higher α- and γ-tocopherol levels in individuals with greater cognitive capacity, although no direct association with cognitive function was statistically confirmed [51]. Tanprasertsuk et al. (2021) found that higher α-tocopherol levels in early-affected brain regions were associated with lower NFT counts and were higher in individuals with Braak stage I-II. However, no significant relationship was observed between α-tocopherol and Mini-Mental State Examination (MMSE) scores [12]. Similarly, Kim et al. identified that individuals with higher levels of β-γ-tocopherol combination levels, particularly in men, non-drinkers and non-smokers, showed improvements in their cognitive function [16].

In contrast, several studies found no significant association. Chan et al. (2024) found no direct association between α-tocopherol stereoisomers and premortem cognitive function [19]. Several studies also reported no significant association between γ-tocopherol levels and cognitive performance [51,57].

In patients with Alzheimer’s disease, lower plasma levels of α-, β-, γ-, and δ-tocopherol, as well as tocotrienols, were associated with poorer cognitive function and elevated oxidative stress markers [55]. Meanwhile, Huang et al. (2018) noted that subjects affected by mild cognitive impairment (MCI) had higher α-tocopherol levels and α-tocopherol/retinol ratios compared to cognitively normal individuals. However, it was the higher α-tocopherol/retinol ratio that was associated with poorer memory and executive function [58]. Lastly, Kuchan et al. have shown that the infant brain showed a preference towards naturally occurring tocopherol compared to synthetic [20]. The summary of the findings is tabulated in Table 4.

#### 3.6.2. Intervention and Dietary Assessment

Intervention and dietary assessment studies have shown varied results regarding the impact of vitamin E supplementation on cognitive performance. Several studies reported improvements in memory, executive function, attention, and processing speed following vitamin E supplementation [38,50,53]. Remington et al. (2015) found that α-tocopherol supplementation enhanced overall cognitive function, reduced the risk of cognitive decline, and stabilized cognitive function in patients with dementia [64]. Nolan et al. (2022) similarly observed slower cognitive decline, improved cognitive category scores, and reduced dementia severity in individuals receiving α-tocopherol as an intervention [2].

However, other studies found no significant cognitive benefits of vitamin E supplementation. Kryscio et al. (2017) reported no differences in dementia incidence, memory function, executive function, learning, or processing speed [18]. Sano et al. (2016) also found no significant effect on cognitive decline, memory, vocabulary, behavior, or dementia progression [61]. Similarly, Li et al. (2015) [8] and Dysken et al. (2014) [68] observed no clinically significant differences in global cognitive function, memory, attention, or orientation, although Dysken et al. (2014) noted a slight, statistically insignificant slowing of cognitive decline. Some studies also found a reduction in cognitive performance associated with vitamin E supplementation, such as Bentsen et al. (2017), who reported a decline in high PUFA patients [59].

Dietary assessment through a survey further supports the potential cognitive benefits of vitamin E intake. Zhang et al. (2023) found that higher dietary α-tocopherol intake was linked to improved memory performance, verbal fluency, semantic memory, processing speed, attention, and working memory [45]. Similarly, Liu et al. (2023) reported that higher α-tocopherol intake was associated with better global cognitive function, episodic memory, perceptual speed, and a protective effect on cognitive performance, particularly in APOEε4 carriers [46]. Other studies indicated that a higher intake of vitamin E was associated with a reduced risk of dementia and Alzheimer’s disease [49,60], as well as better overall cognitive function [46,47]. Beydoun et al. (2020) found that high dietary vitamin E intake slowed cognitive decline and improved verbal memory and working memory, although no significant differences were observed in psychomotor speed or executive function [56]. Paganini-Hill et al. (2023) reported that individuals with higher vitamin E intake had lower odds of developing Alzheimer’s disease neuropathology and were less likely to experience cognitive impairment and dementia, with similar benefits observed across different dosage levels [10]. However, some studies reported no significant effects of vitamin E intake on cognitive status or brain volume reduction [54], and others found that δ-tocopherol did not provide protective cognitive effects in APOEε4 carriers [46]. A summary of the findings is presented in Table 5.

### 3.7. Neurodegenerative Diseases and Neuroprotection

The included studies highlight the potential role of vitamin E in neurodegeneration and neuroprotection across various neuro-related diseases, including Alzheimer’s disease, dementia, Parkinson’s disease, schizophrenia, psychosis, and epilepsy (Table 6). Among all the diseases, AD is the most studied in relation to the effect of vitamin E on brain health.

#### 3.7.1. Alzheimer’s Disease

Supplementation with vitamin E has been shown to reduce AD neuropathology risk [10]. It was found that lower amyloid plaque burden in the frontal and temporal cortex correlated with higher levels of natural RRR-α-tocopherol, while synthetic RSS-α-tocopherol was associated with increased plaques [19]. Another form of tocopherol, γ-tocopherol, also lowered amyloid plaque burden when present in higher concentrations [1,9]. Interestingly, according to a study by Morris et al. (2015), when γ-tocopherol levels were low, higher α-tocopherol concentrations were associated with increased amyloid plaque burden [9]; however, α-tocopherol levels when present independently showed no association with Braak staging or neuritic plaque counts in any brain region [9,12].

Tau pathology, characterized by hyperphosphorylation and aggregation of tau protein, is a major hallmark of AD. A significant correlation was observed between higher synthetic RSS-α-tocopherol levels and increased neurofibrillary tangles (NFTs) in the frontal cortex, temporal cortex, and amygdala [19]. However, the natural form of tocopherol, RRR-α-tocopherol showed no such association. On the other hand, higher γ-tocopherol levels were associated with reduced NFT severity [1,9]. α-Tocopherol was also linked to lower NFT accumulation in brain regions affected in early Braak stages, such as the hippocampus, amygdala, and entorhinal cortex but not in the later-affected brain regions [12]

In APOEɛ4 carriers (i.e., individuals with at least one copy of the ɛ4 allele of the apolipoprotein E (APOE) gene, a well-established genetic risk factor for late-onset AD), higher dietary intake of α-tocopherol was significantly associated with a slower rate of cognitive decline [46]. The protective effects of tocopherols and vitamin E on cognitive function remained significant even after adjusting for APOEɛ4 status [1,10]. However, several studies reported no significant interaction between APOEɛ4 status and tocopherol levels in predicting amyloid or tau pathology [9] or modifying the relationship between vitamin E levels and neurofibrillary tangle counts [12].

The studies also examined molecular markers associated with neuroprotection. Lower α-tocopherol concentrations were associated with reduced expression of key microRNAs (miRNAs), including miR-122, miR-9, miR-21, miR-29b, and miR-132, which are significantly downregulated in AD patients [13]. Additionally, higher brain γ-tocopherol levels were linked to elevated presynaptic protein concentrations in the midfrontal cortex, including SNARE (Soluble NSF Attachment Protein Receptor) complex proteins and syntaxin/SNAP-25 (Synaptosomal-Associated Protein 25 kDa). These proteins play essential roles in synaptic vesicle docking and neurotransmitter release, suggesting that γ-tocopherol may contribute to maintaining synaptic integrity in the AD brain [7]. From an anti-inflammatory perspective, higher tocopherol levels were associated with reduced activated microglia density, supporting an anti-inflammatory environment in the brain [9]. Furthermore, AD subjects exhibited lower tocopherol and tocotrienol levels and higher oxidative/nitrosative damage, with nitrosative stress being particularly linked to neurodegeneration in individuals with longer leukocyte telomere lengths, suggesting a role in cellular aging [13].

#### 3.7.2. Dementia

Vitamin E’s role in dementia appears to vary across different populations and dementia subtypes. In individuals with Down syndrome, vitamin E supplementation did not slow the progression of dementia or cognitive decline [46]. However, in the general aging population, lower dietary vitamin E intake (<13.20 mg/day) was linked to a significantly higher incidence of dementia. In contrast, higher intake (>23.63 mg/day) was associated with a reduced risk of dementia and slower cognitive decline, indicating a potential protective effect [29]. Notably, γ-tocopherol maintained a significant association with synaptic integrity even in individuals with cerebral infarcts, highlighting its relevance for vascular dementia [7]. Moreover, this association remained unaffected in the presence of Lewy body-related pathology, suggesting that vitamin E, particularly γ-tocopherol, may support synaptic health across diverse forms of dementia [7].

#### 3.7.3. Parkinson’s Disease

In Parkinson’s disease, higher dietary vitamin E intake was linked to a lower risk of developing the disease [44] but did not significantly impact disease severity [11]. Additionally, supplementation with Neuroaspis PLP10™ delayed motor symptom progression and reduced Levodopa dose requirements, suggesting a role for vitamin E in disease modulation [52].

#### 3.7.4. Other Neuro-Related Diseases

Vitamin E supplementation was also investigated in psychiatric and seizure-related conditions. In schizophrenia, combining EPA with vitamins E and C was found to reduce psychotic symptoms and improve sustained attention, showing potential synergistic effects on cognitive and behavioral outcomes [59]. However, in another study, although PUFA-E supplementation led to higher α-tocopherol levels and changes in glutathione status, it did not produce significant improvements in psychotic symptoms or PANSS scores during the 12-week intervention period [63]. In epilepsy, vitamin E supplementation led to a significant reduction in seizure frequency compared to placebo and showed notable improvements in EEG patterns [4].

## 4. Discussion

This scoping review explores the current body of literature on Vitamin E, tocopherol and tocotrienol and their effect on human brain health over the past decade. Demographically, half of the studies (*n* = 42) originated from North America (48%), indicating the region’s high research interest in this area. Europe accounted for 29%, Asia 21% and mixed countries (Oceania, Europe, and North America) contributed 2%. Regarding the type of study conducted, 43% are cross-sectional, followed by randomized controlled trials (36%) and cohort studies (19%). Most cross-sectional and cohort studies originated from North America. These studies used established longitudinal cohort databases such as The Chicago Health and Aging Project (CHAP), The Healthy Aging in Neighborhoods of Diversity across the Life Span (HANDLS) study, PREADVISE, Canadian Study of Health and Aging (CSHA), Rush Memory and Aging Project (MAP), Georgia Centenarian Study (GCS), and National Health and Nutrition Examination Survey (NHANES). These cohorts on the aging population provide rich datasets that enable in-depth investigation into dietary patterns and their long-term impacts on brain health of the North American population.

For the past decade, the number of studies utilizing tocopherol and its isomeric forms on human brain health is higher compared to tocotrienol, with α-tocopherol being the most studied isomer (*n* = 22). This can be attributed to the retention ability and higher biological activity of α-tocopherol compared to the other isomers. However, for the last 10 years, only a few studies have been performed on the effect of tocotrienol on human brain health. Around two studies focused solely on tocotrienol [3,38], and another two studies investigated tocotrienol isomers (α-, β-, γ-, and δ-) [13,55]. Gopalan et al. studied the protective activity of mixed tocotrienols in humans with white matter lesions (WMLs). Meanwhile, Sekikawa and colleagues evaluated the effects of combined astaxanthin and tocotrienol intake on cognitive function in healthy adults experiencing memory decline [38]. Two other studies investigated all types of vitamin E including tocotrienol to study the following: (1) the inflammatory markers and microRNA expression in elderly individuals with Alzheimer’s disease in comparison to healthy controls (HC) [13], and (2) to explore the relationship between vitamin E forms and leukocyte telomere length (LTL) in AD patients [55].

On the other hand, only two studies reported on the natural vs. synthetic Vitamin E effects on brain health. Chan and colleagues quantified the α-tocopherol stereoisomers in the temporal cortex of 47 centenarians and correlated them with amyloid plaques and neurofibrillary tangles in 7 different regions [19]. The naturally occurring stereoisomer, RRR-α-tocopherol, was the dominant form of α-tocopherol in the temporal cortex (TC) of all tested subjects. While there was no clear link between TC αT stereoisomers and the centenarians’ cognitive decline prior to death, RRR-αT and RSS-αT showed different patterns in relation to brain aging and neuropathological conditions. Higher levels of RRR-α-tocopherol were associated with fewer plaques in frontal and temporal cortex, whereas higher levels of synthetic RSS-αT were linked to more of those markers of brain aging, including structural changes, i.e., NFTs in the frontal cortex, total cortex, and amygdala [19].

On the other hand, a study by Kuchan et al. (2016) reveals that, in the infant brain, RRR-α-tocopherol was the predominant stereoisomer of α-tocopherol in the frontal cortex (FC), hippocampus (HPC) and visual cortex (VC), despite infants’ extensive exposure to the various stereoisomers in the synthetic all-rac-α-tocopherol [20]. Human infants are born with low levels of α-tocopherol; therefore, doses of vitamins are obtained from human milk or through commercially available infant formulas. Despite exposure to both types of tocopherols, the brain during development prefers the natural tocopherol over the synthetic one [20]. These studies have shown the brain has a preference in retaining natural tocopherol, probably due to the selective binding and transport of RRR-α-tocopherol by α-Tocopherol transfer protein (α-TTP), limiting the circulation of synthetic stereoisomers and other isomers of vitamin E [20].

### 4.1. The Effects of Tocotrienol or Tocopherol Supplementation on Brain Health

#### 4.1.1. Vitamin E Influences Neuroinflammation and Oxidative Stress

The first domain for brain-health indicators in the present scoping review are neuroinflammation and oxidative stress. Neuroinflammation and oxidative stress are two interlinked pathological processes that play a significant role in the onset and progression of numerous neurological disorders. Neuroinflammation happens in response to injury, infection, or abnormal protein accumulation in the brain. While acute inflammation may be protective, chronic activation leads to the release of pro-inflammatory cytokines, chemokines, and reactive oxygen species (ROS), contributing to neuronal damage. Oxidative stress arises when the production of ROS exceeds the brain’s antioxidant defense capacity, resulting in lipid peroxidation, protein oxidation, and DNA damage [69]. Both processes disrupt neuronal integrity and synaptic function, exacerbating conditions such as Alzheimer’s disease, Parkinson’s disease, epilepsy, and schizophrenia. The interest in antioxidants such as vitamin E is growing due to their ability to neutralize ROS, reduce lipid peroxidation, and modulate inflammatory signaling pathways, making them potential therapeutic agents in neuroprotection.

Among the 42 articles reviewed, 12 investigated the effects of vitamin E on neuroinflammation and oxidative stress in humans. Over the past decade, numerous studies have investigated the relationship between neuroinflammation, oxidative stress, and vitamin E. There are several modes of action of vitamin E on inflammatory response that have been mentioned in the articles. Among them are the following: (1) antioxidant action [1,4,51,55,57,59,63,66,67], (2) modulation of cell signaling pathway [13,70,71], (3) gene regulation [13,72,73], (4) inhibition of lipid peroxidation and membrane preservation [66], (5) mitochondrial protection [74], and (6) immune cell boosting [1,75].

Neuroinflammation, a response to harmful stimuli, is mediated by glial cells, particularly microglia and astrocytes. Microglial activation studies by Leeuw et al. (2020) showed that increased brain α- and γ-tocopherol levels were associated with a reduction in activated microglial density, especially in cortical regions [1]. This finding shows the potential of tocopherol in modulating neuroinflammation by influencing glial cell activity. Upon activation, these microglia and astrocytes may secrete a variety of pro-inflammatory cytokines and chemokines that can contribute to the neuroinflammation. However, prolonged inflammation and exposure to oxidative stress may cause harm to the brain. Elevated levels of inflammatory markers are frequently observed in neurodegenerative conditions.

In a study by Boccardi et al. (2023) [13], it was found that individuals with Alzheimer’s disease demonstrated significantly lower levels of α-tocopherol compared to healthy controls, independent of age and gender [32]. Among the inflammatory molecules examined, G-CSF, GM-CSF, IFN-α2, IL-3, and IL-8 were elevated, while IL-17 was decreased in this AD group, highlighting a distinct pro-inflammatory profile of AD with low tocopherol levels. Additionally, they also investigated the exosomal expression of miRNAs including miR-9, miR-21, miR-29b, miR-122, and miR-132 and found that it was significantly downregulated in AD patients. These microRNAs appear to play a critical role in amplifying neuroinflammation and accelerating neurodegeneration in Alzheimer’s disease, as these miRNAs are key modulators of inflammatory signaling, amyloidogenic processing, synaptic plasticity, and neuronal survival [76,77,78,79,80]. Their expression may result in the overactivation of glial cells, dysregulation of enzymes like BACE1 and ADAM10, and impaired clearance of amyloid-β, hence worsening the pathological hallmarks of AD [76,77]. Additionally, miR-122 levels were significantly correlated with peripheral cytokines, including GM-CSF, IFN-α2, IL-1α, IL-8, and MIP-1β, suggesting a link between miRNA dysregulation and inflammation in AD [32]. The study also reported that lower α-tocopherol levels were associated with AD, and that miR-122 levels partially mediated this relationship, implying that vitamin E may exert its neuroprotective effects via epigenetic regulation of inflammatory pathways [13].

Additionally, oxidative stress markers provide further insight into the protective function of vitamin E. The group receiving vitamin E supplementation showed higher total antioxidant capacity (TAC) [4,57,59,66], increased catalase activity [4], modulated glutathione levels [4,63] and, in some cases, reduced SOD activity [66]. Homocysteine, another neuroinflammatory marker, showed different findings in relation to vitamin E. One study reported lower homocysteine levels in healthy volunteers receiving a combination treatment that included vitamin E [66]. However, another study investigating the relationship between physical activity, total homocysteine, and vitamin E in the elderly found no significant correlation between vitamin E and total homocysteine levels [51].

Several findings suggest that vitamin E plays a crucial role in maintaining cellular redox balance by neutralizing reactive oxygen species (ROS) and reducing lipid peroxidation. Smesny et al. (2015) demonstrated that vitamin E also influences the inhibition of phospholipase A2, preventing excessive lipid peroxidation and maintaining membrane stability. The ability of α-tocopherol to regulate oxidative stress and inflammation suggests that it serves as a crucial neuroprotective factor, particularly in aging and neurodegeneration [63]. Casati et al. (2020) observed that elevated α-tocopherylquinone/α-tocopherol and 5-nitro-γ-tocopherol/γ-tocopherol ratios indicated increased oxidative and nitrosative stress, particularly in individuals with lower total tocopherol and tocotrienol levels [55].

Overall, vitamin E exerts its effects through a combination of direct antioxidant activity, regulation of inflammatory cytokines, suppression of microglial activation, and modulation of lipid metabolism. Its role in maintaining redox homeostasis, preventing lipid peroxidation, and preserving cellular integrity highlights its importance in neuroprotection. These findings underscore the need for optimal vitamin E levels to support brain health, particularly in aging populations where oxidative stress and neuroinflammation become increasingly detrimental.

#### 4.1.2. Vitamin E’s Ability to Protect the Brain Structure and Function

Brain structure and function form the biological foundation of all cognitive, emotional, and behavioral processes. Structurally, the brain is composed of various interconnected regions, including the cerebral cortex, hippocampus, amygdala, basal ganglia, and cerebellum, which are responsible for specific neurological and physiological functions. Functionally, these regions coordinate to regulate activities such as memory formation, sensory processing, motor control, and emotional regulation. Optimal brain function depends on the integrity of neuronal networks, synaptic connectivity, and the balance of neurotransmitters. Disruptions in these systems, whether due to neurodegeneration, inflammation, or oxidative damage, can impair cognitive performance and contribute to neurological disorders. Maintaining the structure and function of the brain requires adequate nutrient supply, protection from oxidative stress, and efficient cellular repair mechanisms. Vitamin E, a potent lipid-soluble antioxidant, plays a crucial role in maintaining membrane stability, preventing lipid peroxidation, and promoting overall brain health.

**Figure 4:**
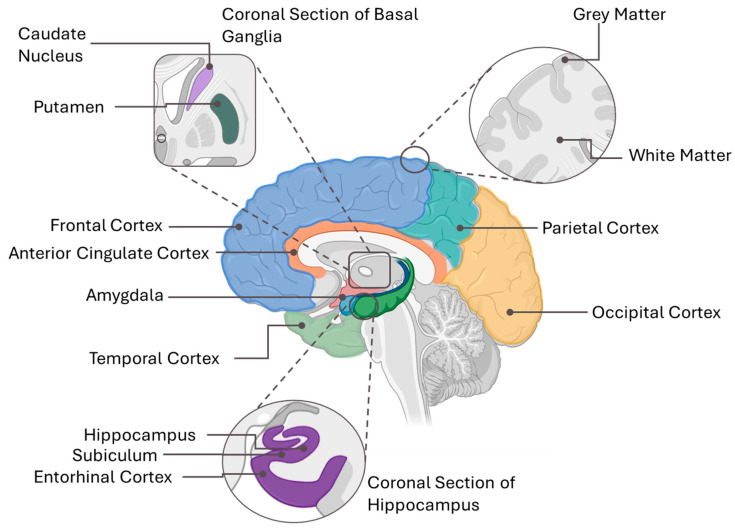
Vitamin E distribution and its potential neuroprotective effects in the brain. Region highlights with color other than grey shows key brain regions affected by vitamin E, including the anterior cingulate cortex, basal ganglia (caudate nucleus and putamen), hippocampus (with coronal subregions: subiculum, entorhinal cortex), and amygdala. These regions are implicated in cognitive, emotional, and motor functions, and are particularly vulnerable to oxidative stress, underlining the significance of vitamin E antioxidant role in maintaining structural and functional brain integrity.

Across studies, α-tocopherol consistently emerges as the dominant form in the brain, compared to the other types of vitamin E. Additionally, the brain’s preference for the natural form of vitamin E is evident in the frontal cortex, hippocampus, and visual cortex, where Kuchan et al. (2016) found RRR-α-tocopherol concentrations to be significantly higher than those of synthetic forms [20]. The frontal cortex, which is critical for executive function and decision-making, exhibited the highest α-tocopherol retention (10.5 μg/g), suggesting a potential role of vitamin E in cognitive performance. The hippocampus, which is essential for memory and learning, had slightly lower α-tocopherol levels (6.8 μg/g), yet it still retained a selective preference for the natural form, reinforcing the idea that vitamin E supports neuronal function by mitigating oxidative damage and preserving synaptic plasticity. The visual cortex, with the lowest α-tocopherol concentration (5.5 μg/g), suggests that brain regions with higher metabolic activity and greater oxidative burden may have a higher demand for vitamin E. In contrast, sensory-processing areas rely less on its neuroprotective effects (Figure 4) [20,81].

Beyond its role in regional vitamin E distribution, α-tocopherol has been implicated in preventing NFT accumulation. Tanprasertsuk et al. (2021) reported an inverse association between α-tocopherol levels and NFT counts in early-affected brain regions, including the amygdala, entorhinal cortex, hippocampus, and subiculum, regions highly involved in memory processing [12]. This suggests that vitamin E may play a protective role in slowing tau pathology in the initial stages of neurodegeneration, potentially through its antioxidant and anti-inflammatory properties. However, in later-affected neocortical regions, such as the frontal and temporal cortices, α-tocopherol did not show significant associations with NFT accumulation, indicating that its protective effects may diminish as the disease progresses. Interestingly, α-tocopherol levels were not correlated with neuritic plaque (NP) counts, suggesting that its neuroprotective mechanisms may be more relevant to tau pathology than amyloid-β accumulation [12].

At the synaptic level, γ-tocopherol plays a more significant role than α-tocopherol in preserving neurotransmission and synaptic integrity. Leeuw et al. (2020) found that higher γ-tocopherol levels in the midfrontal cortex were associated with increased presynaptic protein concentrations, including SNARE proteins, complexin-I, complexin-II, synaptotagmin, and synaptophysin composite [7]. This suggests that γ-tocopherol contributes to maintaining synaptic function and connectivity, potentially offering protection against cognitive decline. However, these associations were region-specific, as no significant effects were observed in the inferior temporal cortex. Notably, α-tocopherol did not show any significant correlation with presynaptic protein levels in either region, further emphasizing the distinct roles of different vitamin E isomers. This regional specificity suggests that γ-tocopherol may be particularly important for preserving synaptic integrity in brain areas vulnerable to oxidative stress, such as the frontal cortex, which participates in higher cognitive processing.

The distribution of vitamin E across cortical and subcortical regions further reinforces its structural significance. Leeuw et al. (2020) found that cortical α-tocopherol levels (232.2 pmol/mg) were significantly higher than those in the subcortical regions (158.7 pmol/mg), suggesting a greater demand for vitamin E in areas responsible for executive function and cognition. Conversely, subcortical γ-tocopherol levels (63.6 pmol/mg) were slightly higher than cortical levels (57.1 pmol/mg), raising the possibility that different vitamin E isomers serve distinct neuroprotective functions depending on brain region [1]. Additionally, vitamin E appears to modulate neuroimmune responses, as higher α- and γ-tocopherol levels were associated with reduced microglial activation in cortical regions but showed no significant effects in subcortical areas. Given that microglial activation is a major contributor to neuroinflammation and neurodegeneration, these findings suggest that vitamin E may be more effective in controlling inflammatory responses in cortical regions rather than deeper brain structures.

Structural imaging studies provide additional insights into vitamin E’s role in white and grey matter integrity. Dorey et al. (2023) found that tocopherol concentrations were significantly lower in both white and grey matter of Alzheimer’s disease brains compared to healthy controls, with a more pronounced deficiency in white matter. Since white matter integrity is essential for efficient neural communication, these findings suggest that vitamin E depletion may contribute to structural vulnerability and cognitive decline [48]. Similarly, Gopalan et al. (2014) [3] demonstrated that tocotrienol supplementation significantly reduced the progression of white matter lesions (WMLs), with negligible changes (−1.9%) in the tocotrienol group compared to a 23.3% increase in WML volume in the placebo group. This suggests that tocotrienols, rather than tocopherols alone, may play a unique role in preserving white matter structure and preventing demyelination. The differential effects of vitamin E isomers on brain structures highlight the need for a more nuanced understanding of their functions, particularly in relation to myelin integrity and neural efficiency.

Genetic factors may further modulate the structural impact of vitamin E on the brain. Haptoglobin (Hp) is a plasma protein involved in binding free hemoglobin to prevent oxidative damage. The Hp gene exists in three common genotypes (Hp 1-1, Hp 2-1, and Hp 2-2) with different antioxidants and inflammatory properties [82]. Livny et al. (2020) reported a genotype-dependent effect of vitamin E intake on brain volume, where Hp 2-1 carriers exhibited increased inferior frontal gyrus volume with higher vitamin E intake, whereas Hp 1-1 carriers showed the opposite trend. This suggests that individual genetic differences may influence how vitamin E contributes to structural resilience, potentially affecting its neuroprotective efficacy across populations [54]. Additionally, Paganini-Hill et al. (2023) investigated brain weight as a general measure of neurodegeneration and found no significant difference between groups with low and high Alzheimer’s pathology, suggesting that brain weight alone may not be a reliable indicator of vitamin E’s impact on structural integrity [10].

Collectively, these findings highlight the multifaceted role of vitamin E in brain structure and function, with α-tocopherol exhibiting a stronger influence on early tau pathology and γ-tocopherol playing a crucial role in synaptic preservation. The preferential retention of α-tocopherol in cortical regions, particularly the frontal cortex and hippocampus, suggests a role in cognitive resilience, while γ-tocopherol’s effects on synaptic integrity highlight its importance in neurotransmission. The decline of tocopherol levels in white matter, coupled with the protective effects of tocotrienols against white matter lesion progression, further suggests that different vitamin E isomers contribute to distinct structural and functional outcomes. These structural modifications are intrinsically linked to cognitive function, as the integrity of neuronal networks, synaptic efficiency, and neuroprotection against degeneration directly influence memory, executive function, and overall cognitive performance. Given these connections, it becomes crucial to explore how vitamin E modulates cognitive abilities, including its role in processing speed, attention, verbal fluency, and memory retention, to better understand its impact on maintaining brain function and delaying cognitive decline.

#### 4.1.3. Vitamin E Delays Cognitive Decline

Cognitive performance refers to an individual’s ability to acquire, process, store, and retrieve information, encompassing domains such as memory, attention, executive function, language, and visuospatial skills. It is influenced by a wide range of factors including age, education, lifestyle, genetic predisposition, and underlying neurological health. Decline in cognitive performance is often one of the earliest signs in various neurodegenerative and neuropsychiatric disorders, such as Alzheimer’s disease, dementia, schizophrenia, and epilepsy [16,51,56,83]. At the molecular level, cognitive function is closely tied to synaptic plasticity, neuronal integrity, and neurotransmitter balance, all of which can be disrupted by oxidative stress, inflammation, and lipid peroxidation [7,26,51]. Cognitive performance emerged as the most frequently studied domain across the reviewed literature, reflecting the key role of cognitive decline in age-related neurodegenerative conditions. However, despite the volume of research, findings remain inconsistent, proving the complexities of vitamin E’s role in cognitive health.

In this scoping review, to better interpret the findings on cognitive performance, the articles were divided into two groups: vitamin E level-based assessments and intervention/dietary intake studies. These divisions were performed according to methodological differences. Level-based studies, often cross-sectional, reveal associations between circulating or brain vitamin E concentrations and cognitive outcomes, offering insights into potential mechanisms but are limited by their inability to establish causality [12,13,51,55]. In contrast, intervention and dietary studies provide stronger causal interpretation by evaluating the effect of long-term intake or supplementation. However, their outcomes vary depending on isomer, dosage, baseline status, and population characteristics [2,18,45,46].

Observational studies assessing vitamin E levels generally indicate that higher circulating or brain concentrations of α-tocopherol correlate with improved cognitive performance [13,51,57]. These associations are most prominent in domains such as executive function, memory, and daily living skills. Notably, Tanprasertsuk et al. [12] found reduced neurofibrillary tangles in early-stage Alzheimer’s brain regions with higher α-tocopherol levels, suggesting a possible role in delaying tau pathology. However, the absence of correlation with MMSE scores [12] and inconsistent findings regarding premortem cognition [19] raise concerns about the sensitivity of cognitive assessments and potential effects in these studies. The cognitive benefits of γ- and δ-δ-tocopherol are still unclear. While some benefits are observed in non-smokers and non-drinkers [16], their general effect on cognitive function is minimal or non-significant in many studies [51,57,63]. This may reflect their different antioxidant properties or the limitation in interactions with α-tocopherol transport protein at the cellular level. Nonetheless, the observed lower amount of various tocopherol and tocotrienol isomers in Alzheimer’s patients might suggest that neuroprotection should involve all vitamin E isomers, rather than the action of individual isomers [55].

Tocotrienols, though less frequently studied, may have potential, particularly given their unique distribution in the brain and stronger antioxidant activity. The lack of clinical studies directly comparing tocotrienol and tocopherol isomers in populations with cognitive impairment represents a significant research gap.

Intervention and supplementation trials yielded inconsistent results. Several studies suggest cognitive benefits from α-tocopherol, particularly in memory, executive function, and processing speed [2,38,53,64]. However, a significant proportion of RCTs report no benefit [8,18,61,68] or even cognitive worsening in subpopulations with specific dietary backgrounds, such as high PUFA intake [59]. According to Bentsen et al. (2017), vitamin E supplementation alone impaired sustained attention in individuals with high baseline PUFA levels, likely due to excessive increases in serum α-tocopherol which may have acted as a pro-oxidant in an already lipid-rich environment, thereby disrupting redox balance and contributing to cognitive decline [59]. In the end, these inconsistencies highlight the importance of context such as the baseline of vitamin E status, duration of supplementation, coexisting nutrient profiles, and disease stage which are able to modulate the efficacy of vitamin E interventions. Another possible reason is the widespread use of synthetic vitamin E (all-rac-α-tocopherol) in many trials. Evidence indicates that natural RRR-α-tocopherol is preferentially retained in tissues and may be more biologically active [2,64]. Additionally, very few studies incorporate genetic stratification, despite emerging evidence that APOEε4 carriers may respond differently to vitamin E interventions [46].

Dietary intake surveys generally support a protective effect of long-term vitamin E consumption, with several studies reporting slower cognitive decline, reduced dementia risk, and better memory and attention scores in individuals with higher intake [10,45,46,47,49,60]. However, causality cannot be inferred from these associations due to confounding lifestyle variables and reverse causation.

Taken together, the evidence suggests that vitamin E, particularly α-tocopherol, plays a role in maintaining cognitive performance. However, its clinical utility remains uncertain due to heterogeneity in outcomes, possible isomer-specific effects, genetic interactions, and methodological variability across studies. Future trials should employ stratified designs, consider bioactive isomers, and explore combination therapies that may show synergies with the antioxidant and possible neuroprotective properties of vitamin E.

#### 4.1.4. Vitamin E Protect Against Neurodegenerative Diseases

Vitamin E’s role in neuroprotection and neurodegenerative diseases is supported by its effects on oxidative stress, neuroinflammation, synaptic integrity, and disease pathology across multiple conditions, including Alzheimer’s disease, dementia, Parkinson’s disease, and epilepsy.

1. Alzheimer’s Disease

Alzheimer’s disease is a progressive neurodegenerative disorder characterized by cognitive decline, memory loss, and behavioral changes [84]. This debilitating disease is marked by the accumulation of amyloid-β plaques and neurofibrillary tangles composed of hyperphosphorylated tau protein [84,85], and its occurrence could be worsened by oxidative stress, neuroinflammation, mitochondrial dysfunction, and synaptic loss [1,7,13]. The severity of tau pathology in AD, is usually assessed using Braak staging, a neuropathological classification that traces the spread of neurofibrillary tangles from the transentorhinal region in early stages (I–II), to the limbic areas (III–IV), and finally to the neocortex in the most advanced stages (V–VI) [86].

In Alzheimer’s disease, vitamin E has been extensively investigated for its potential role in reducing amyloid and tau pathology, two key hallmarks of the disease. Higher levels of RRR-α-tocopherol have been associated with a lower amyloid plaque burden in the frontal and temporal cortices, while the synthetic RSS-α-tocopherol isomer has been linked to increased plaque accumulation, suggesting that the stereoisomer composition of α-tocopherol may influence its neuroprotective potential [19]. Additionally, γ-tocopherol levels were negatively correlated with the amyloid plaque burden, reinforcing its role as a neuroprotective agent [1,9]. However, when γ-tocopherol levels were low, higher α-tocopherol concentrations were associated with increased plaque accumulation, implying that an imbalance between vitamin E isomers may influence AD pathology [9]. These findings suggest that while vitamin E may have protective effects, the specific isomer and its availability could determine its efficacy in reducing amyloid burden. Interestingly, α-tocopherol levels were not independently associated with Braak staging or neuritic plaque (NP) counts [9,12], indicating that its role may be more significant in early disease stages rather than in the progression of widespread neurodegeneration.

Similarly, the relationship between vitamin E and tau pathology highlights its complex role in neurodegeneration. Higher RSS-α-tocopherol levels correlated with increased NFT accumulation in the frontal cortex, temporal cortex, and amygdala, while no significant association was found between RRR-α-tocopherol and NFT formation [44]. However, γ-tocopherol levels were inversely associated with NFT severity [1,9], suggesting that γ-tocopherol, rather than α-tocopherol, may play a protective role against tau pathology. Additionally, higher α-tocopherol levels were linked to lower NFT counts in brain regions affected during the early stages of AD, including the amygdala, hippocampus, entorhinal cortex, and subiculum, but showed no significant association in later-affected regions such as the frontal, temporal, and parietal cortices [10,12]. This suggests that while α-tocopherol may help delay early-stage tau pathology, it does not prevent its widespread accumulation in later disease stages. These findings align with broader research indicating that vitamin E’s neuroprotective effects may be most pronounced in the early phases of neurodegeneration when oxidative stress and neuroinflammation are more manageable [68].

The potential neuroprotective role of vitamin E extends beyond its direct interaction with amyloid and tau pathology. Higher brain γ-tocopherol levels were significantly associated with increased presynaptic protein levels, including SNARE proteins, Complexin I and II, Syntaxin/SNAP-25, and Synaptotagmin, particularly in the midfrontal cortex [7]. These proteins play a critical role in neurotransmission and synaptic stability, suggesting that vitamin E, particularly γ-tocopherol, may help preserve synaptic integrity and reduce neurodegenerative synaptic loss. This aligns with findings that lower α-tocopherol levels are associated with downregulated miRNA expression, including miR-122, miR-9, miR-21, miR-29b, and miR-132, all of which are implicated in neuroinflammation and neuronal repair mechanisms [13]. The interplay between vitamin E, synaptic proteins, and miRNA regulation further supports the hypothesis that vitamin E acts at multiple levels to maintain neuronal health.

Genetic factors, particularly the APOɛ4 genotype, have been considered in studies examining vitamin E’s neuroprotective effects. While APOɛ4 is a major genetic risk factor for AD, the protective effects of tocopherols and vitamin E remained significant even after adjusting for APOɛ4 status [1,10], suggesting that vitamin E exerts its benefits independently of genetic risk. Higher α-tocopherol intake was associated with a significantly slower rate of cognitive decline in APOE4 carriers [46], reinforcing the idea that adequate vitamin E levels may mitigate some of the genetic risks associated with neurodegeneration. However, no significant interaction was found between APOɛ4 status and tocopherol levels in predicting amyloid or tau pathology, indicating that while vitamin E may support cognitive resilience, its effects may not be strong enough to counteract APOɛ4-related neuropathological changes [9,12].

2. Dementia

Beyond AD, vitamin E has been investigated for its broader role in dementia. Dementia is a syndrome that causes interference with daily life and independence. There are a few types of dementia, each with different underlying pathologies, such as vascular dementia, Lewy body dementia, frontotemporal dementia, and mixed dementia, but AD is the most common cause of dementia.

Lower dietary vitamin E intake (<13.20 mg/day) was associated with a significantly higher incidence of dementia, while higher intake (>23.63 mg/day) was linked to a reduced risk and slower cognitive decline [29]. These findings suggest that maintaining adequate vitamin E levels through diet may offer protective effects against general cognitive deterioration. Additionally, γ-tocopherol’s association with synaptic integrity remained significant even in individuals with infarcts [7], indicating potential benefits for vascular dementia. Furthermore, vitamin E was found to protect synapses even in individuals with Lewy body-related dementia, reinforcing its potential role in multiple dementia subtypes [7]. Interestingly, in specific populations such as individuals with Down syndrome, vitamin E supplementation did not prevent the progression of dementia symptoms, underscoring the need for population-specific approaches [61].

3. Parkinson’s disease

Parkinson’s disease (PD) is a progressive neurodegenerative disorder affecting motor control causing tremor bradykinesia, rigidity, and postural instability. In addition to motor symptoms, patients may experience cognitive decline, depression, sleep disturbances, and autonomic dysfunction. In Parkinson’s disease (PD), higher dietary vitamin E intake has been associated with a lower risk of developing the disease [44], although it does not appear to influence disease severity [11]. The protective effects may be linked to vitamin E’s ability to reduce oxidative stress and neuroinflammation, both of which contribute to dopaminergic neuronal loss in PD. However, factors such as age, hypertension, and cardiovascular disease (CVD) were stronger predictors of PD risk than vitamin E intake alone [44], suggesting that while vitamin E may be beneficial, its effects are likely influenced by broader metabolic and vascular factors. Clinical trials using vitamin E formulations such as Neuroaspis PLP10™ significantly delayed PD motor symptom progression and reduced the need for increased levodopa doses [52], suggesting that targeted formulations may enhance the effectiveness of vitamin E in specific neurological conditions.

4. Other Neurological Disorders

Vitamin E has also been studied in psychiatric and seizure-related conditions, including schizophrenia, psychosis, and epilepsy. These disorders, while distinct in clinical presentation, often share underlying mechanisms such as oxidative stress and mitochondrial dysfunction.

In schizophrenia, combined supplementation of EPA and vitamins E and C was found to reduce psychotic symptoms and improve sustained attention [59], suggesting potential benefits in managing oxidative stress-related psychiatric disorders. Similarly, PUFA-E supplementation led to increased α-tocopherol levels and altered glutathione metabolism in psychotic patients [63], though it did not significantly impact symptom severity. In epilepsy, vitamin E supplementation significantly reduced seizure frequency and improved EEG findings compared to placebo [4], suggesting improved neuronal stability and reduced excitotoxic damage.

**Figure 5:**
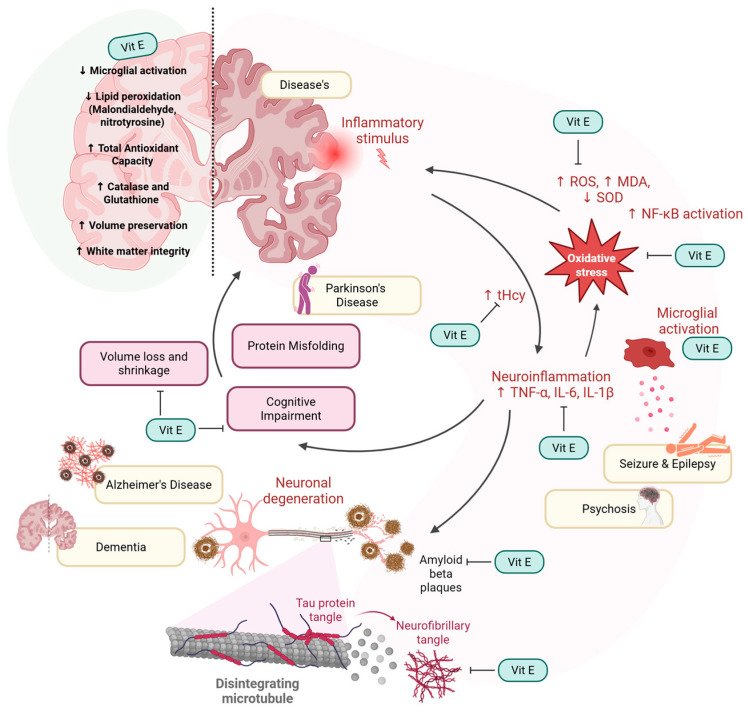
Proposed mechanistic pathway illustrating the role of Vitamin E in modulating neuroinflammation, oxidative stress, and neurodegeneration. Inflammatory stimuli initiate a cascade involving increased reactive oxygen species (ROS), elevated pro-inflammatory cytokines (e.g., TNF-α, IL-6, IL-1β), oxidative damage, and microglial activation. These processes contribute to neuronal damage, white matter loss, hippocampal atrophy, and ultimately cognitive impairment. Downstream consequences include protein misfolding, tau pathology, and amyloid plaque deposition, which collectively drive neurodegenerative diseases such as Alzheimer’s and Parkinson’s disease. Vitamin E acts at multiple levels of this pathway, reducing oxidative stress (↓ MDA, ↑ catalase and glutathione), inhibiting inflammatory signaling (↓ NF-κB activation), attenuating microglial activation, preserving brain volume and white matter integrity, and supporting cognitive performance.

Taken together, the evidence presented in this scoping review highlights the multifaceted roles of vitamin E in preserving brain health (Figure 5). The evidence suggests that vitamin E plays an important role in neuroprotection, with its effects varying based on factors. Given that neurodegeneration is a progressive process involving multiple pathways, future research should explore how vitamin E supplementation, dietary intake, and lifestyle interventions can be optimized to maintain brain health. Understanding how vitamin E interacts with emerging neuroprotective strategies, including anti-inflammatory therapies, antioxidant-based treatments, and lifestyle modifications, will be crucial in determining its role in promoting long-term brain health and resilience against neurodegenerative diseases.

### 4.2. Other Possible Factors and Confounding Factors

Tocotrienol and tocopherol supplementation have been explored for their potential effects on brain health; however, several confounding factors may influence their efficacy. Lifestyle factors such as smoking, alcohol consumption, and physical activity levels may also contribute to variations in brain-health outcomes. Additionally, factors such as age, BMI and baseline nutritional status play a crucial role in vitamin E metabolism, affecting its bioavailability and overall effectiveness [9,87].

The method of supplementation is another critical factor for Vitamin E supplementation. Variation in intake methods can influence absorption and bioavailability such as timing of intake, i.e., in the morning or evening, and intake as a food companion, i.e., taken together with or without food [88]. In a controlled clinical study by Carroll and Schade, vitamin E (800 IU of natural RRR-α-tocopherol) was administered either before breakfast or before a high-fat evening meal in individuals with type 2 diabetes. The results demonstrated that vitamin E taken before the high-fat supper was significantly more effective in dampening the postprandial rise in systemic inflammation marker’s C-reactive protein (CRP) compared to both no supplementation and prebreakfast dosing [88]. Understanding optimal timing and method could enhance clinical recommendations for maximizing vitamin E efficacy.

The appropriate dosage of tocotrienol and tocopherol remains a key research question. While some studies have focused on high doses, others have investigated lower, physiologically relevant doses. Standardized guidelines must be developed based on long-term safety and efficacy data. The Joint Expert Committee on Food Additives (JECFA) has defined an acceptable daily intake (ADI) of 0.15–2.0 mg/kg body weight per day for α-tocopherol [89]. However, other types of isomers and tocotrienols have not yet been standardized. On the other hand, research comparing tocotrienols and tocopherols suggests that tocotrienols might require lower dosages for effective neuroprotection compared to tocopherols, primarily due to their enhanced ability to cross the blood–brain barrier and their greater antioxidative potential against neuroinflammatory markers [90,91,92].

### 4.3. How Do Tocotrienols and Tocopherols Compare in the Maintenance of Brain Health?

To date, a conclusive comparison between tocopherols and tocotrienols in terms of brain-health maintenance is limited. This is primarily due to the lack of clinical trials investigating tocotrienols, despite convincing evidence from in vitro and animal studies suggesting their superior neuroprotective potential. Tocopherols have been extensively studied in human populations and incorporated into randomized clinical trials; however, tocotrienols have not yet achieved similar clinical validation. Because of that, comparison between tocopherol and tocotrienol in preserving cognitive function, modulating neuroinflammation, and preventing neurodegenerative diseases in humans remains difficult. Nonetheless, the efforts in researching tocopherols and tocotrienols’ effect on brain health should not be taken for granted.

Tocopherol’s neuroprotective activity in humans has been discussed extensively in this scoping review due to it being widely studied in the last decade. It is well known that α-tocopherol plays a crucial role in maintaining neuronal health due to its antioxidant and anti-inflammatory properties. Numerous clinical and observational studies have linked higher α-tocopherol status with slower cognitive decline, reduced oxidative damage, reduced early-stage tau pathology, and preservation of brain volume, particularly in elderly populations [9,12,14,16,20,27,29,57]. Its ability to scavenge free radicals and inhibit lipid peroxidation has been demonstrated across various models of neurodegeneration, including Alzheimer’s disease, Parkinson’s disease, and cerebral ischemia [9,44,65].

On the other hand, tocotrienols have demonstrated potency in reducing oxidative stress and lipid peroxidation in the brain, preserving mitochondrial function, and reducing AD pathology in cell and animal models [35,83,93]. Despite these promising findings, tocotrienols have their limitations in translation to clinical practice, due to their lower bioavailability and rapid metabolism, which are caused by their poor affinity to α-TTP [22,23]. Upon ingestion, tocotrienol maximum concentration peaked at 4 h after supplementation and then reduced significantly after 24 h compared to tocopherol that has better retention in the body [94]. Moving forward, retention of tocotrienol should be investigated to better understand its pharmacokinetics and optimize its therapeutic window. This short half-life limits its ability to sustain in the brain, especially for chronic neurodegenerative conditions where long-term antioxidant presence may be crucial.

While this review provides a broad overview of the available evidence, several limitations must be considered. The included studies exhibited significant heterogeneity in design, supplementation methods, and outcome measures, making it difficult to arrive at a definite conclusion. Variability in dosage, duration, and the form of supplementation further complicates the ability to compare the efficacy of different vitamin E isomers. Additionally, the absence of long-term data limits the strength of these findings. As a result, the research questions regarding the comparative effects of tocotrienols and tocopherols on cognitive performance, neuroinflammation, oxidative stress, and brain structure remain only partially answered. Future studies with more standardized methodologies, long-term follow-up, and subgroup analyses are needed to clarify these issues.

### 4.4. Future Directions: Optimizing Vitamin E Delivery and Efficacy for Brain Health

The vitamin E absorption mechanisms, especially tocotrienol, should be further studied. Despite tocotrienol’s lower bioavailability in plasma serum, the distribution of tocotrienol in selective tissues such as the lung, skin and brain has been noticed [95]. In a study by Khanna et al. (2005), it was shown that oral supplementation of α-tocotrienol to infertile female α-TTP knock-out mice restored fertility even under α-tocopherol deficiency, which suggests the possibility of other mechanisms for tocotrienol transportation and absorption besides α-TTP [96]. Investigating the absorption mechanisms of tocotrienol will provide a clearer understanding of tocotrienol distribution, hence maximizing its therapeutic potential.

Additionally, there is a need for comparative studies on different formulations and delivery methods to optimize vitamin E absorption and clinical effectiveness. Innovations such as encapsulation techniques, nano formulations, and diet-based interventions should be explored. One delivery approach that can be implemented is the use of Self-Emulsifying Drug Delivery Systems (SEDDSs). SEDDSs are designed to enhance the oral bioavailability of poorly water-soluble drugs by enhancing their solubility and passive permeability, hence maximizing the initial absorption [97,98]. A study has shown that using SEDDS to deliver tocotrienol formulations improves oral bioavailability in human subjects [99]. Another drug delivery system besides SEDDS is nanocarrier systems. Liposomes, Nanostructured lipid carriers (NLCs), solid lipid nanoparticles (SLNs), and nanoemulsions are among the nanometer-scale carriers that can be used to encapsulate vitamin E or tocotrienol. They are designed to have properties that help in their absorption into the body by enhancing the drug solubility, controlled degradation and facilitated targeted delivery to specific tissues [98,100,101,102].

An additional approach to maximizing the health benefits of vitamin E is through co-supplementation with other nutrients or compounds. A 12-week study of co-supplementation of tocotrienol with astaxanthin to Japanese adults experiencing memory decline showed significant improvement in their composite and verbal memory without any adverse effect [38]. Other examples of co-supplementation with other nutrients include Omega-3 fatty acids, coenzyme Q10 and vitamin C [2,50,84]. It has been shown that co-supplementation with these nutrients enhances cognitive function and offers neuroprotective benefits, thus highlighting the need for future investigation. However, the competitive interaction between the co-supplementation should also be considered. It was shown that in animal and human studies, co-supplementation of tocopherols and tocotrienols appears to induce a competitive interaction that generally favors α-tocopherol uptake at the expense of tocotrienols [103]. Hence, long-term studies on how co-supplementation of tocopherol and tocotrienol impacts tissue distribution, storage, and their efficacy in neuroprotective effects in the brain should be considered. Clarifying these interactions will optimize vitamin E supplementation strategies for general health and help design approaches in tocotrienol delivery to target tissues (especially the brain).

Vitamin E metabolites (from both tocopherols and tocotrienols) have unique bioactivities, including anti-inflammatory, neuroprotective, antioxidant, and anticancer effects, which are, in some cases, more potent than their parent compounds [104]. The carboxyethyl-hydroxychroman metabolite of γ-tocopherol (γ-CEHC), carboxychromanols (e.g., 5′-carboxymethylbutyl hydroxychroman and the 13′-carboxychromanol (13′-COOH)) has been identified to have anti-inflammatory properties and display potent bioactivity [105,106]. Investigating the effect of these metabolites may reveal new strategies for leveraging vitamin E metabolites in neurodegenerative and other chronic diseases.

The gut–brain axis is an emerging area of interest, with evidence suggesting that vitamin E may impact gut microbiota composition, which could subsequently affect neuroinflammation and cognitive function [37]. It is crucial to explore how these microbiota changes translate into cognitive benefits, particularly through immune modulation, the production of short-chain fatty acids, and the maintenance of gut barrier integrity. Well-designed clinical and preclinical studies that incorporate metagenomic, metabolomic, and inflammatory profiling are needed to elucidate these interactions. Understanding this bidirectional relationship may lead to new therapeutic strategies for neurodegenerative and neuroinflammatory conditions, utilizing targeted vitamin E interventions.

**Figure 6:**
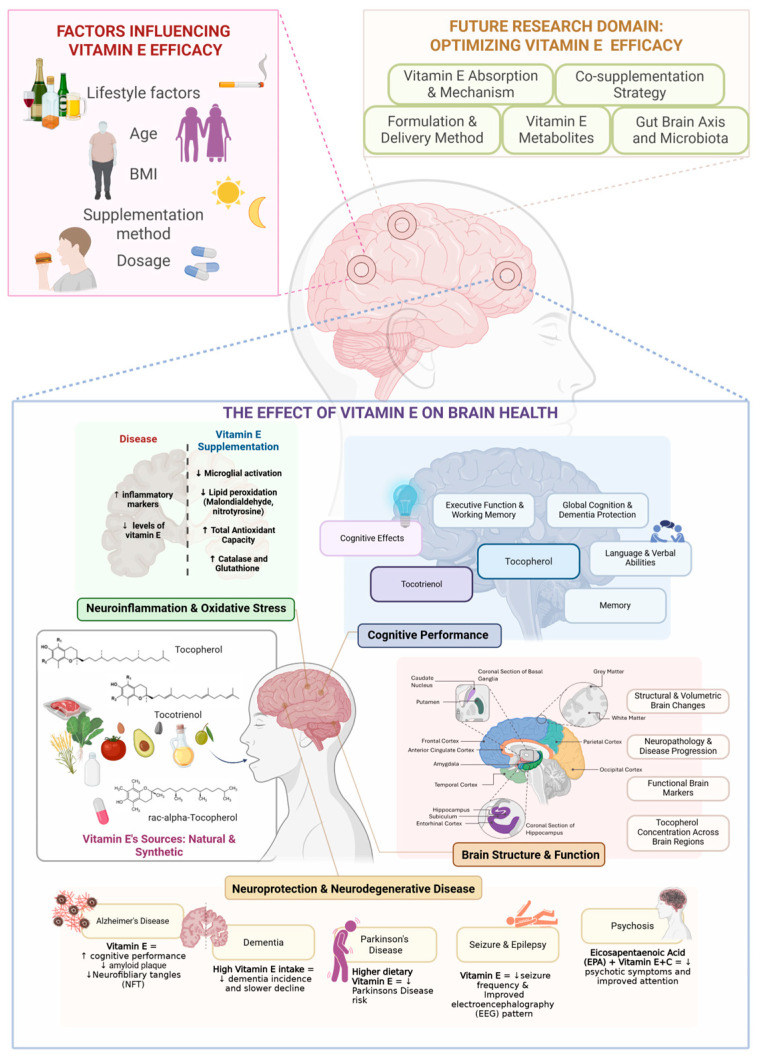
Summary of vitamin E’s role in brain health. This figure illustrates the interconnected effects of tocopherols and tocotrienols across key domains of brain health, including cognitive performance, brain structure, neuroinflammation, oxidative stress, and neurodegenerative diseases. Additionally, factors such as age, APOE genotype, and baseline vitamin E status may influence absorption and efficacy. Future studies should further investigate vitamin E absorption mechanisms, delivery strategies, and long-term outcomes.

Finally, current research on tocotrienol and tocopherol supplementation is limited by small sample sizes and variability in study design. More well-designed RCTs with larger cohorts from various regions worldwide are required to establish their impact on brain health. Many existing studies assess tocopherol’s short-term outcomes, highlighting the need for longitudinal research with extended follow-up periods to evaluate its long-term effects on cognitive performance, neuroinflammation, and the progression of neurodegenerative diseases. Future research should also integrate genetic and lifestyle factors that may influence vitamin E’s effects, allowing for the development of personalized supplementation strategies. Addressing these research gaps will contribute to a more comprehensive understanding of vitamin E’s role in brain health, leading to evidence-based supplementation guidelines and improved clinical recommendations (Figure 6).

## 5. Conclusions

This scoping review highlights the multifaceted roles of tocopherol and tocotrienol in maintaining brain health, particularly in the domains of cognitive performance, neuroinflammation, oxidative stress, and structural preservation over the last 10 years. While α-tocopherol remains the most extensively studied and widely retained form of vitamin E in human tissues, emerging evidence underscores the distinct effects of tocotrienols. Tocotrienols demonstrate promising anti-inflammatory and antioxidative capacities, particularly in preserving white matter integrity and modulating neurodegenerative pathways. Despite these findings, inconsistencies in study outcomes, variations in assessment methods, and the limited number of long-term clinical trials complicate the ability to make a definitive conclusion. Future research should focus on well-designed, population-specific trials that investigate the synergistic effects between tocopherol and tocotrienol isomers, including their dose–response relationships and the influence of genetic modifiers. Additionally, as emphasized in this review, optimizing delivery systems (e.g., SEDDS, nanocarriers), evaluating co-supplementation strategies, and exploring the bioactivity of vitamin E metabolites are critical next steps. Long-term randomized controlled trials tailored to specific populations are also needed to assess efficacy across various cognitive domains. Furthermore, incorporating multi-omics approaches and examining gut–brain interactions may yield novel insights into the neuroprotective and cognitive-enhancing potential of vitamin E. As the global burden of cognitive decline and neurodegenerative diseases continues to rise, expanding our understanding of these compounds may inform future nutritional strategies on how to support cognitive longevity and brain resilience.

## Figures and Tables

**Table 1:** Summary of studies examining the relationship between vitamin E and brain-health outcomes over the past 10 years. Studies are categorized by design, population age, vitamin E form and assessment method, study focus, brain-health domain(s), and outcome measures.

Source (Year)	Study Design	Age Group (Years)	Vitamin E Form	Vitamin E Assessment Method	Primary Study Focus	Brain-Health Outcomes Measured	Brain-Health Themes
Chan et al. (2024) [19]	Cross-sectional	100+	α-tocopherol, γ-tocopherol	Survey	Amyloid plaques; Neurofibrillary tangles (NFT)	NFTs and amyloid plaque count Global Deterioration Scale (GDS)-dementia APOEɛ genotype	Brain Structure and Function Cognitive Performance Neuroprotection and Neurodegenerative Diseases
Hao et al. (2024) [44]	Cross-sectional	40+	Vitamin E	Survey	Parkinson’s disease	Parkinson’s disease prevalence (measured through anti-Parkinson medication use)	Neuroprotection and Neurodegenerative Diseases
Zhang et al. (2023) [45]	Cross-sectional	65+	α-tocopherol,	Survey	Cognitive performance	CERAD (Consortium to Establish a Registry for Alzheimer’s Disease) test. Animal Fluency Test (AFT) Digit Symbol Substitution Test (DSST) Patient Health Questionnaire-9 (PHQ-9)	Cognitive Performance
Liu et al. (2023) [46]	Cohort	65+	α-tocopherol, β-tocopherol, δ-tocopherol, γ-tocopherol	Survey	Cognitive performance; APOE ε4 allele	APOEɛ4 genotype (assessed via SNPs rs7412 and rs429358) Episodic memory performance Perceptual speed Mini-Mental State Examination (MMSE) score	Cognitive Performance Neuroprotection and Neurodegenerative Diseases
Li et al. (2023) [47]	Cross-sectional	60+	Vitamin E	Survey	Cognitive performance	CERAD-WL (Word Learning Test) CERAD-DR (Delayed Recall Test) Animal Fluency Test (AF) Digit Symbol Substitution Test (DSST)	Cognitive Performance
Dorey et al. (2023) [48]	Cross-sectional	56+	α-tocopherol, δ-tocopherol, γ-tocopherol	Brain extract	Alzheimer’s disease	Levels of antioxidants (tocopherol) Distribution of analytes across brain regions.	Brain Structure and Function Neuroprotection and Neurodegenerative Diseases
Boccardi et al. (2023) [13]	Cross-sectional	70+	α-tocopherol, β-tocopherol, δ-tocopherol, γ-tocopherol, α-tocotrienol, β-tocotrienol, δ-tocotrienol, γ-tocotrienol	Blood	miRNAs; Alzheimer’s disease; inflammation	Exosomal miRNA levels: let-7f-5p, miR-9, miR-15a, miR-21, miR-29b, miR-122, miR-132, miR-29a, miR-128, miR-491, miR-146, miR-34, and miR-874. Inflammatory cytokines: EGF, EOTAXIN, G-CSF, GM-CSF, INF-α2, IFN-γ, IL-1RA, IL-1α, IL-1β, IL-2, IL-3, IL-4, IL-5, IL-6, IL-7, IL-8, IL-10, IL-12p40, IL-12p70, IL-13, IL-15, IL-17, IP-10, MCP-1, MIP-1α, MIP-1β, TNF-α, TNF-β, VEGF, and RANTES. Mini-Mental State Examination (MMSE) Clinical Dementia Rating (CDR) Geriatric Depression Scale (GDS) Activities of Daily Living (ADL) Instrumental Activities of Daily Living (IADL)	Neuroinflammation and Oxidative Stress Cognitive Performance Neuroprotection and Neurodegenerative Diseases
Zhang et al. (2023) [49]	Cross-sectional	60+	Vitamin E	Survey	Cognitive performance	CERAD Word Learning Test Animal Fluency Test (AFT) Digit Symbol Substitution Test (DSST)	Cognitive Performance Neuroprotection and Neurodegenerative Diseases
Paganini-Hill et al. (2023) [10]	Cohort	90+	Vitamin E	Survey; brain autopsies	Alzheimer’s disease; amyloid plaques; Neurofibrillary tangles	Mini-Mental State Examination (MMSE) scores Dementia diagnosis based on DSM-IV criteria. Amyloid plaques (Thal Phase scoring) Neurofibrillary tangles (Braak Stage scoring) Neuritic plaque density (CERAD criteria) Brain weight Presence of APOE ε4 allele (genetic risk factor for AD) Presence of APOE ε2 allele (potential protective factor) Vascular neuropathology	Brain Structure and Function Cognitive Performance Neuroprotection and Neurodegenerative Diseases
Liu et al. (2023) [46]	Cohort	60+	Vitamin E	Survey	Dementia; cognitive performance	Mini-Mental State Examination (MMSE) Auditory verbal learning test Trail Making Tests A and B Conflicting Instructions Task (Go/No Go Task) Stick Test Modified Common Objects Sorting Test Modified Fuld Object Memory Evaluation Incident Dementia Cases—Diagnosis based on DSM-IV criteria. APOE ε4 Genotype Shanghai Cognitive Activities Scale. Center for Epidemiologic Studies Depression Scale (CES-D)	Neuroprotection and Neurodegenerative Diseases
Power et al. (2022) [50]	Randomized Controlled Trial	65+	α-tocopherol (RRR-α-tocopherol) [as a part of the mixture of intervention]	Administered	Cognitive performance;	Serum concentration of Vitamin E (α-tocopherol) Macular Pigment Optical Volume (MPOV) Reaction Time (RTI) Macular Pigment Density (MPOV) Montreal Cognitive Assessment (MoCA) Repeatable Battery for the Assessment of Neuropsychological Status (RBANS) Cambridge neuropsychological test automated battery (CANTAB)	Cognitive Performance Neuroprotection and Neurodegenerative Diseases
Nolan et al. (2022) [2]	Randomized Controlled Trial	79+	α-tocopherol (RRR-α-tocopherol) [as a part of the mixture of intervention]	Administered	Alzheimer’s disease	Serum vitamin E (D-α-tocopherol) Cognitive function (Mini-Mental State Examination, MMSE) Dementia Severity (Dementia Severity Rating Scale, DSRS) Quality of life assessment (self-reported and caregiver-reported) Frailty assessment (Clinical Frailty Scale)	Neuroprotection and Neurodegenerative Diseases
Alghadir et al. (2021) [51]	Cross-sectional	56–81	α-tocopherol, γ-tocopherol	Blood	Cognitive performance; oxidative stress	Loewenstein Occupational Therapy Cognitive Assessment (LOTCA) Mini-Mental State Examination (MMSE) Serum vitamin E Total Antioxidant Capacity (TAC) Nitric Oxide (NO) Levels Total Homocysteine (tHcy) Levels	Neuroinflammation and Oxidative Stress Cognitive Performance Neuroprotection and Neurodegenerative Diseases
Tanprasertsuk et al. (2021) [12]	Cross-sectional	98+	α-tocopherol, γ-tocopherol	Brain	Amyloid plaques; Neurofibrillary tangles; Alzheimer’s disease	Neurofibrillary tangles (NFT) counts Neuritic plaques (NP) counts Braak and Braak NFT Staging Presence of APOE ε4 allele Mini-Mental State Examination (MMSE) scores	Neuroinflammation and Oxidative Stress Brain Structure and Function Cognitive Performance Neuroprotection and Neurodegenerative Diseaseas
Pantzaris et al. (2021) [52]	Randomized Controlled Trial	40–75	Vitamin E, γ-tocopherol	Administered	Parkinson’s disease	Hoehn and Yahr Staging Scale Unified Parkinson’s Disease Rating Scale (UPDRS III)	Neuroprotection and Neurodegenerative Diseases
Stavrinou et al. (2020) [53]	Randomized Controlled Trial	65+	Vitamin E, γ-tocopherol	Administered	Cognitive performance	Sit-to-Stand Tests (STS-5, STS-30, STS-60) 6-Minute Walk Test (6MWT) Handgrip Strength (HGS) Timed-Up-and-Go (TUG) test. Addenbrooke’s Cognitive Examination-Revised (ACE-R) Mini-Mental State Examination (MMSE) Trail Making Test (TMT) Stroop Color and Word Test (STROOP) Symbol Cancelation Test Short Form-36 Health Survey (SF-36) Fatigue Severity Scale Pittsburgh Sleep Quality Index Epworth Sleepiness Scale	Cognitive Performance Neuroprotection and Neurodegenerative Diseases
Sekikawa et al. (2020) [38]	Randomized Controlled Trial	40+	Tocotrienol	Administered	Cognitive performance	Subjective memory symptoms questionnaire Mood assessment (Likert scale) Anxiety-related symptoms Cognitrax Composite Memory Verbal Memory (Immediate and Delayed Recall) Visual Memory Processing Speed Executive Function Reaction Time Cognitive Flexibility Complex Attention Social Acuity Working Memory Sustained Attention Motor Speed Brain-Derived Neurotrophic Factor (BDNF)	Cognitive Performance
Livny et al. (2020) [54]	Cross-sectional	65+	Vitamin E, α-tocopherol, β-tocopherol, δ-tocopherol, γ-tocopherol	Survey	Haptoglobin	Haptoglobin (Hp) genotype (Hp 1-1, Hp 2-1, Hp 2-2) Inflammatory markers (IL-6, C-reactive protein) White matter hyperintensities (WMH) Brain atrophy (gray matter volume) Total intracranial volume (TICV) Mini-Mental State Examination (MMSE) Neuropsychological assessment (detailed cognitive battery) Cognitive status classification (normal, MCI, dementia)	Brain Structure and Function Cognitive Performance Neuroprotection and Neurodegenerative Diseases
Leeuw et al. (2020) [1]	Cross-sectional	88.5 ± 6.0	α-tocopherol, γ-tocopherol	Survey; brain autopsies	Microglia	Brain Tocopherol Levels (α-tocopherol, γ-tocopherol) Microglia Density (total, activated, macrophages) Amyloid Load (percent area occupied by amyloid β) Neurofibrillary Tangle (NFT) Severity (Braak staging) Inflammation Markers (activated microglia stages I/II/III) Genetic Marker: APOE ε4 Allele (presence vs. absence) Clinical Diagnosis of Alzheimer’s Disease (AD)	Neuroinflammation and Oxidative Stress Brain Structure and Function Neuroprotection and Neurodegenerative Diseases
Leeuw et al. (2020) [7]	Cross-sectional	88.5 ± 6.0	α-tocopherol, γ-tocopherol	Survey; brain autopsies	Presynaptic protein	Presynaptic protein levels (SNARE protein composite, synaptotagmin synaptophysin composite, syntaxin, SNAP-25, complexin-I, complexin-II, septin-5) Protein–protein interactions (syntaxin/SNAP-25 and SNAP-25/syntaxin) Alzheimer’s disease pathology markers (Amyloid plaques, Neurofibrillary tangles) Lewy body pathology Cerebral infarcts	Brain Structure and Function Neuroprotection and Neurodegenerative Diseases
Casati et al. (2020) [55]	Cross-sectional	70+	Vitamin E, Tocopherol, α-tocopherol, β-tocopherol, δ-tocopherol, γ-tocopherol, Tocotrienol, α-tocotrienol, β-tocotrienol, δ-tocotrienol, γ-tocotrienol	Blood	Alzheimer’s disease	Oxidative/Nitrosative stress markers Leukocyte Telomere Length (LTL) as a marker of cellular aging Amyloid-β(Aβ), tau, and p-tau levels Neuroimaging Data Mini-Mental State Examination (MMSE). Clock Drawing Test (CDT). Neuropsychological tests: Trail Making Test, verbal fluency, digit span forward/backwards, verbal learning, Token Test, Rey’s figure copy and delayed recall, Raven Colored Progressive Matrices. Geriatric Depression Scale (GDS). Hand Grip Strength (physical performance measure).	Neuroinflammation and Oxidative Stress Cognitive Performance Neuroprotection and Neurodegenerative Diseases
Beydoun et al. (2020) [56]	Cohort	30–65	Vitamin E	Survey	Cognitive performance	Mini-Mental State Examination (MMSE) California Verbal Learning Test (CVLT): List A and Delayed Free Recall (DFR) Benton Visual Retention Test (BVRT) Digit Span Forward (DS-F) Digit Span Backwards (DS-B) Animal Fluency (AF) Brief Test of Attention (BTA Clock Drawing Test (CDT) Trail Making Test (TRAILS A and B) Centre for Epidemiological Studies Depression Scale (CES-D)	Cognitive Performance
Schirinzi et al. (2019) [11]	Case–control	60+	Vitamin E	Survey	Parkinson’s disease	Neurodegenerative Disease Risk (PD prevalence)	Neuroprotection and Neurodegenerative Diseases
Tanprasertsuk et al. (2018) [57]	Cross-sectional	98+	α-tocopherol, γ-tocopherol	Blood; brain	Cognitive performance	Brain Region Analysis (Frontal Cortex, Temporal Cortex) Oxidative Stress and Inflammation Indicators Neuroprotective Markers Global Deterioration Scale (GDS)	Brain Structure and Function Cognitive Performance
Kim et al. (2018) [16]	Cross-sectional	60–79	Vitamin E, α-tocopherol, γ-tocopherol	Blood	Cognitive performance	Mini-Mental State Examination for Dementia Screening (MMSE-DS)	Cognitive Performance Neuroprotection and Neurodegenerative Diseases
Huang et al. (2018) [58]	Cross-sectional	55–80	α-tocopherol, γ-tocopherol	Blood; survey	Cognitive performance; ApoE	Montreal Cognitive Assessment [MoCA] Apolipoprotein E (ApoE) Genotype	Cognitive Performance Neuroprotection and Neurodegenerative Diseases
Bentsen et al. (2017) [59]	Randomized Controlled Trial	18–39	Vitamin E	Administered	Schizophrenia	Oxidative Stress Continuous Performance Test (CPT-IP) Hopkins Verbal Learning Test Letter-Number Span Test Paced Auditory Serial Addition Test (PASAT) Stroop Test Verbal Fluency Test Positive and Negative Syndrome Scale (PANSS) Psychotic Symptoms Severity (Positive Subscale)	Neuroinflammation and Oxidative Stress Cognitive Performance Neuroprotection and Neurodegenerative Diseases
Kryscio et al. (2017) [18]	Cohort	60+	Vitamin E	Administered	Alzheimer’s disease	APOE ε4 carrier status Memory Impairment Screen (MIS) Modified Telephone Interview for Cognitive Status (TICS-m) CERAD neuropsychological battery (during RCT phase) New York University Paragraph Delayed Recall Ascertain Dementia 8-Item Informant Questionnaire (AD8)	Cognitive Performance Neuroprotection and Neurodegenerative Diseases
Basambombo et al. (2017) [60]	Cohort	65+	Vitamin E	Survey	Alzheimer’s disease; cognitive performance	Diagnostic and Statistical Manual of Mental Disorders (DSM-III-R). Modified Mini-Mental State (3MS) Examination. National Institute of Neurological and Communicative Disorders and Stroke and the Alzheimer’s Disease and Related Disorders Association (NINCDSADRDA) Cognitive impairment, not dementia (CIND)	Cognitive Performance Neuroprotection and Neurodegenerative Diseases
Sano et al. (2016) [61]	Randomized Controlled Trial	50+	Vitamin E	Administered	Cognitive performance; Down syndrome; dementia	APOE genotype analysis Brief Praxis Test (BPT) (primary measure for cognitive function and dementia progression) Fuld Object Memory Test (verbal and visual memory) New Dot Test (memory and cognitive function) Vocabulary Test Orientation Test Dementia diagnosis using DSM-IV and ICD-9 criteria. Adverse event (AE) and serious adverse event (SAE) tracking Depressive symptoms and crying (AE tracking) Behavioral function assessment Dementia medication usage (Memantine, Donepezil)	Cognitive Performance Neuroprotection and Neurodegenerative Diseases
Kuchan et al. (2016) [20]	Cross-sectional	5 to 488 days old	α-tocopherol	Brain	Level of Vitamin E in infant brain	Regional differences in α-tocopherol distribution Total α-tocopherol concentration in different brain regions	Brain Structure and Function Cognitive Performance Neuroprotection and Neurodegenerative Diseases
Ding et al. (2016) [62]	Cohort	60+	Vitamin E	Administered	Alzheimer’s disease; dementia; sleep apnea	APOE ɛ4 genotype (genetic risk factor for dementia) Memory Impairment Screen (MIS) CERAD-e battery TICS-m (Modified Telephone Interview for Cognitive Status) AD8 Dementia Screening Instrument	Neuroprotection and Neurodegenerative Diseases
Mehvari et al. (2016) [4]	Randomized Controlled Trial	20–50	Vitamin E	Administered	Seizure; epilepsy	EEG analysis Oxidative Stress and Antioxidant Markers Seizure Frequency Types of Seizures (Focal, Generalized) Response to Antiepileptic Drugs (AEDs) Effect of Vitamin E Supplementation on Seizure Control	Neuroinflammation and Oxidative Stress Brain Structure and Function Neuroprotection and Neurodegenerative Diseases
Smesny et al. (2015) [63]	Randomized Controlled Trial	13–25	Vitamin E, α-tocopherol, δ-tocopherol, γ-tocopherol	Administered	Psychosis; oxidative stress;	Positive and Negative Syndrome Scale (PANSS total score) Global Assessment of Functioning (GAF) score Oxidative Stress marker	Neuroinflammation and Oxidative Stress Neuroprotection and Neurodegenerative Diseases
Remington et al. (2015) [64]	Randomized Controlled Trial	65.9 ± 11.3	α-tocopherol	Administered	Cognitive performance	Cognitive function scores (Dementia Rating Scale—DRS) Executive function performance (CLOX-1) Presence of Mild Cognitive Impairment (MCI)	Cognitive Performance Neuroprotection and Neurodegenerative Diseaseas
Remington et al. (2015) [65]	Randomized Controlled Trial	77.8 ± 8.4	α-tocopherol	Administered	Cognitive performance; mood; Alzheimer’s disease	Dementia Rating Scale (DRS) Clox-1 test (Executive function) Mini-Mental State Examination (MMSE) Memory domain of DRS Behavioral and Psychological Symptoms of Dementia (BPSD) Caregiver-reported Neuropsychiatric Inventory (NPI) Functional independence (Activities of Daily Living (ADL))	Cognitive Performance Neuroprotection and Neurodegenerative Diseases
Muss et al. (2015) [66]	Randomized Controlled Trial	18 to 90	Vitamin E	Administered	Neuroprotective	Antioxidative Capacity (FORT) Oxidative Stress (FORD) Blood–Brain Barrier Integrity Mitochondrial Deficiency Neurodegeneration Indicators	Neuroinflammation and Oxidative Stress Neuroprotection and Neurodegenerative Diseases
Morris et al. (2015) [9]	Cohort	88.5 ± 5.9	α-tocopherol, γ-tocopherol	Brain; Blood; Survey	Alzheimer’s disease	Amyloid plaques (amyloid-β load) Neurofibrillary tangles (Braak staging) Brain region-specific analyses APOE ε4 genotype Clinical Alzheimer’s disease diagnosis	Brain Structure and Function
Li et al. (2015) [8]	Randomized Controlled Trial	60–75	α-tocopherol	Administered	Cognitive performance	Plasma amyloid-β (Aβ) levels Plasma estradiol (E2) Mini-Mental State Examination (MMSE) scores Hasegawa Dementia Scale (HDS)	Cognitive Performance Neuroprotection and Neurodegenerative Diseases
Polidori et a l. (2014) [67]	Cross-sectional	65+	α-tocopherol	Blood	Alzheimer’s disease	Mini-Mental State Examination (MMSE) Oxidative Stress Marker	Neuroinflammation and Oxidative Stress Neuroprotection and Neurodegenerative Diseases
Gopalan et al. (2014) [3]	Randomized Controlled Trial	35+	Tocotrienol	Administered	Neuroprotective; brain	White Matter Lesions (WMLs) Brain Volume Changes Inflammatory Biomarkers	Brain Structure and Function Neuroprotection and Neurodegenerative Diseases
Dysken et al. (2014) [68]	Randomized Controlled Trial	53+	α-tocopherol	Administered	Alzheimer’s disease; cognitive performance	Mini-Mental State Examination (MMSE) Alzheimer’s Disease Assessment Scale-Cognitive Subscale (ADAS-cog) Dependence Scale Caregiver Activity Survey (CAS) Neuropsychiatric Inventory (NPI) Alzheimer’s Disease Cooperative Study/Activities of Daily Living (ADCS-ADL) Inventory Neuropsychiatric Inventory (NPI) (measuring psychiatric and behavioral symptoms) APOE ε4 status Charlson Risk Index score	Cognitive Performance Neuroprotection and Neurodegenerative Diseases

Abbreviations: α-, β-, γ-, δ- refer to isomeric forms of tocopherols and tocotrienols. APOE = Apolipoprotein E; MMSE = Mini-Mental State Examination; MoCA = Montreal Cognitive Assessment; CERAD = Consortium to Establish a Registry for Alzheimer’s Disease; DSST = Digit Symbol Substitution Test; AFT/AF = Animal Fluency Test; PHQ-9 = Patient Health Questionnaire-9; CDR = Clinical Dementia Rating; GDS = Geriatric Depression Scale; ADL = Activities of Daily Living; IADL = Instrumental Activities of Daily Living; RBANS = Repeatable Battery for the Assessment of Neuropsychological Status; CANTAB = Cambridge Neuropsychological Test Automated Battery; ACE-R = Addenbrooke’s Cognitive Examination-Revised; TMT = Trail Making Test; STROOP = Stroop Color and Word Test; LOTCA = Loewenstein Occupational Therapy Cognitive Assessment; BTA = Brief Test of Attention; CVLT = California Verbal Learning Test; BVRT = Benton Visual Retention Test; CLOX-1 = Clock Drawing Executive Function Test; DRS = Dementia Rating Scale; PANSS = Positive and Negative Syndrome Scale; GAF = Global Assessment of Functioning; PASAT = Paced Auditory Serial Addition Test; TICS-m = Modified Telephone Interview for Cognitive Status; MIS = Memory Impairment Screen; AD8 = Ascertain Dementia 8-item Questionnaire; HDS = Hasegawa Dementia Scale; NPI = Neuropsychiatric Inventory; BPSD = Behavioral and Psychological Symptoms of Dementia; TAC = Total Antioxidant Capacity; NO = Nitric Oxide; tHcy = Total Homocysteine; FORT = Free Oxygen Radical Test; FORD = Free Oxygen Radical Defense; BDNF = Brain-Derived Neurotrophic Factor; CRP = C-reactive protein; UPDRS III = Unified Parkinson’s Disease Rating Scale Part III; DSRS = Dementia Severity Rating Scale; CAS = Caregiver Activity Survey; ADCS-ADL = Alzheimer’s Disease Cooperative Study–Activities of Daily Living; NFTs = Neurofibrillary Tangles; WMLs = White Matter Lesions; WMH = White Matter Hyperintensities; TICV = Total Intracranial Volume; Hp = Haptoglobin; AE/SAE = Adverse Event/Serious Adverse Event.

**Table 2:** Summary of included human studies on tocopherol and tocotrienol supplementation and their effects on neuroinflammation and oxidative stress. This table outlines the characteristics of the included studies, including author, year, vitamin E form examined, tested markers, origin of the sample, and the outcomes. ↓: lower; ↑: higher.

Source (Year)	Vitamin E Form	Tested Markers	Sample Origin	Outcomes
Boccardi et al. (2023) [13]	α-tocopherol, β-tocopherol, δ-tocopherol, γ-tocopherol, α-Tocotrienol, β-tocotrienol, δ-tocotrienol, γ-tocotrienols	Cytokines: EGF, EOTAXIN, G-CSF, GM-CSF, INF-α2, IFN-γ, IL-10, IL-12p40 IL-12p70, IL-13, IL-15, IL-17, IL-1RA, IL-1α, IL-1β, IL-2, IL-3, IL-4, IL-5, IL-6, IL-7, IL-8, IP-10, MCP-1, MIP-1, MIP-1α, TNF-α, TNF-β, VEGF, RANTES Exosomal miRNAs: let7f5p, miR-9, miR-15a, miR-21, miR29-b, miR-122, miR-132, miR29-a, miR-128, miR-491, miR-146, miR-34, miR-87	Blood	AD patients: ↓ α-Tocopherol ↑ G-CSF, GM-CSF, INF-α2, IL-3, IL-8 ↓ miR-21, miR29-b, miR-9, miR-122, miR-132 Correlation of miR-122 and plasma level of GM-CSF, INF-α2, IL-1α, IL-8, and MIP-1β Correlation of miR-122 and α-tocopherol and AD (AD: ↓ α-tocopherol, ↓ miR-122) No significant difference in tocotrienol levels between AD and healthy
Alghadir et al. (2021) [51]	α-tocopherol, γ-tocopherol	Total homocysteine (tHcy), Nitric oxide; NO Total oxidant capacity	Blood	Cognitive Decline Patients: ↑ Total Homocysteine (tHcy) ↓ Total oxidant capacity ↑ physical activity = ↓ Total Homocysteine (tHcy) No correlation between vitamin E and tHcy
Leeuw et al. (2020) [1]	α-tocopherol, γ-tocopherol	Microglia activation	Brain tissue	↑ brain α- and γ-tocopherol = ↓ activated microglia density (but not in subcortical brain regions)
Casati et al. (2020) [55]	Vitamin E, Tocopherol, α-tocopherol, β-tocopherol, δ-tocopherol, γ-tocopherol, Tocotrienol, α-Tocotrienol, β-tocotrienol, δ-tocotrienol, γ-tocotrienols	α-Tocopherylquinone/α-Tocopherol Ratio (Oxidative Stress Marker) 5-Nitro-γ-Tocopherol/γ-Tocopherol Ratio (Nitrosative Stress Marker) Leukocyte Telomere Length (LTL)	Blood	AD patients: ↓ α-, β-, γ- and δ-tocopherol, α- and δ-tocotrienol, Total tocopherols, total tocotrienols and total vitamin E ↑ α-tocopherylquinone/α-tocopherol and 5-nitro-γ-tocopherol/γ-tocopherol Telomere length: ↑ γ-Tocopherol, total tocopherols, and total Vitamin E levels, ↑ telomere length
Tanprasertsuk et al. (2018) [57]	α-tocopherol, γ-tocopherol	Level of antioxidants: [α-Tocopherol (αT), γ-Tocopherol (γT), Lutein, Zeaxanthin, Cryptoxanthin, β-Carotene, Lycopene] n-6/n-3 Polyunsaturated Fatty Acid (PUFA) Ratio	Blood; Brain tissue	Centenarians: ↑ α-Tocopherol, Lutein, and β-Carotene, ↑ cognitive performance ↑ n-6/n-3 PUFA, ↓ cognitive performance
Polidori et al. (2014) [67]	α-tocopherol	SOD, GPX	Blood	↓ Vitamin C and E are associated with Carotid Intima-Media Thickness (C-IMT)
Bentsen et al. (2017) [59]	Vitamin E	Serum α-Tocopherol level Total antioxidant status s-F2-Isoprostane Malondialdehyde (MDA)	Blood	High PUFA: ↑ serum α-Tocopherol, ↑ TAS, ↑ DHA levels
Smesny et al. (2015) [63]	Vitamin E, α-tocopherol, δ-tocopherol, γ-tocopherol	Vitamin E level Glutathione markers (GSHt, GSHr and GSSG)	Blood	PUFA-E: ↑ α-Tocopherol ↓ GSHt
Muss et al. (2015) [66]	Vitamin E	Homocysteine (Hcy) Superoxide Dismutase (SOD) Lipid Peroxidation Nitrotyrosine Antioxidative capacity	Blood	Vitamin E: ↓ HCY, SOD, lipid peroxidation, nitrotyrosine ↑ Antioxidative capacity
Mehvari et al. (2016) [4]	Vitamin E	Total Antioxidant capacity (TAC) Catalase Glutathione Malondialdehyde	Blood	Vitamin E: ↑TAC, Catalase, Glutathione

Abbreviations: AD = Alzheimer’s Disease; EGF = Epidermal Growth Factor; EOTAXIN = Eosinophil Chemotactic Protein; G-CSF = Granulocyte-Colony-Stimulating Factor; GM-CSF = Granulocyte-Macrophage Colony-Stimulating Factor; INF-α2/IFN-γ = Interferon-alpha2/Interferon-gamma; IL = Interleukin; IP-10 = Interferon gamma-induced protein 10; MCP-1 = Monocyte Chemoattractant Protein-1; MIP-1α/β = Macrophage Inflammatory Protein-1 alpha/beta; TNF-α/TNF-β = Tumor Necrosis Factor alpha/beta; VEGF = Vascular Endothelial Growth Factor; RANTES = Regulated on Activation, Normal T Cell Expressed and Secreted; miR = microRNA; tHcy/HCY = Total Homocysteine; NO = Nitric Oxide; TAC = Total Antioxidant Capacity; SOD = Superoxide Dismutase; GPX = Glutathione Peroxidase; GSHt/GSHr/GSSG = Total, Reduced, and Oxidized Glutathione; PUFA = Polyunsaturated Fatty Acid; DHA = Docosahexaenoic Acid; MDA = Malondialdehyde; LTL = Leukocyte Telomere Length; α-Tocopherylquinone/5-Nitro-γ-Tocopherol = Oxidative and Nitrosative Stress Markers.

**Table 3:** Summary of vitamin E forms, brain regions, and their associations with structural and functional outcomes. This table presents the relationship between different forms of vitamin E (tocopherol and tocotrienol) and their reported effects on specific brain regions. The table summarizes structural and functional brain outcomes as identified through neuroimaging, biochemical, or cognitive assessments. Where available, the associated anatomical regions (e.g., white matter, hippocampus, cortical areas) and the nature of the effect (e.g., preservation, enhancement, or degeneration) are provided.

Authors (Year)	Vitamin E Form	Outcome
Kuchan et al. (2016) [20]	α-tocopherol	**Level in Infant brain** Frontal Cortex (FC) ↑ Mean RRR-α-tocopherol concentration: 10.5 μg/g Synthetic α-tocopherol concentrations (all-rac-α-tocopherol): RRS: 1.0–1.5 μg/g; RSR: 0.8–1.0 μg/g; RSS: 0.7–0.9 μg/g; Σ2S: 0.2–0.3 μg/g Hippocampus (HPC) ↑ Mean RRR-α-tocopherol concentration: 6.8 μg/g Synthetic α-tocopherol levels: RRS: ~1.0 μg/g; RSR: ~0.9 μg/g; RSS: ~0.8 μg/g; Σ2S: 0.3 μg/g Visual Cortex (VC) ↑ Mean RRR-α-tocopherol concentration: 5.5 μg/g Synthetic α-tocopherol levels: RRS: ~1.0 μg/g; RSR: ~0.8 μg/g; RSS: ~0.7 μg/g; Σ2S: 0.3 μg/g
Tanprasertsuk et al. (2018) [57]	α-tocopherol, γ-tocopherol	**Level in Centenarians** Frontal cortex: α-tocopherol: 8.3 ± 4.1 μg/g γ-tocopherol: 1.2 ± 1.1 μg/g Temporal cortex α-tocopherol: 7.9 ± 3.8 μg/g γ-tocopherol: 1.0 ± 0.9 μg/g
Tanprasertsuk et al. (2021) [12]	α-tocopherol, γ-tocopherol	**Neurofibrillary tangle (NFT) and Neural plaque (NP) In different brain region vs. α-tocopherol, γ-tocopherol** Early-affected regions in Alzheimer’s disease (Braak I–II and III–IV stages): Amygdala (Amy), Entorhinal Cortex (Ent), Hippocampus (Hip, CA1 region), Subiculum (Sub) ↑ α-Tocopherol correlated with lower NFT counts. No significant correlation of α-Tocopherol with NP No significant correlation of γ-Tocopherol with NFT and NP Later-affected regions (Braak V–VI stage): Frontal Cortex (FC, Brodmann Area 9), Temporal Cortex (TC, Brodmann Areas 21–22), Parietal Cortex (PC, Brodmann Areas 39–40) No significant correlation of α-Tocopherol with NFT counts No significant correlation of α-Tocopherol with NP No significant correlation of γ-Tocopherol with NFT and NP Braak III–IV (limbic stage) had lower α-tocopherol levels than those in Braak I-II (trans entorhinal stage)
Leeuw et al. (2020) [7]	α-tocopherol, γ-tocopherol	**Brain tocopherol level vs. presynaptic protein of the elderly** Midfrontal Cortex α-tocopherol: 231.8 pmol/mg not significant γ-tocopherol: 57.8 pmol/mg γ-Tocopherol showed significant positive associations with: SNARE protein composite, complexin-I, Complexin-II, Synaptotagmin-synaptophysin composite, Septin-5 Inferior Temporal Cortex α-tocopherol: 172.9 pmol/mg not significant γ-tocopherol: 56.7 pmol/mg not significant
Leeuw et al. (2020) [1]	α-tocopherol, γ-tocopherol	**Brain tocopherol level vs. activated microglia of the elderly.** Cortical tocopherol: α-tocopherol: 232.2 pmol/mg (IQR: 76.0–356.4) γ-tocopherol: 57.1 pmol/mg (IQR: 42.9–92.5) Cortical microglia density: Stage I/II/III (Total Count): 153.2 cells/mm^2^ (IQR: 111.2–194.0) Stage II/III (Activated): 3.9 cells/mm^2^ (IQR: 1.2–11.7) Stage III (Macrophage-like): 0.8 cells/mm^2^ (IQR: 0.1–2.9) Subcortical: α-tocopherol: 158.7 pmol/mg (IQR: 105.0–286.6) γ-tocopherol: 63.6 pmol/mg (IQR: 48.4–81.3) Subcortical microglia density: Stage I/II/III (Total Count): 209.2 cells/mm^2^ (IQR: 175.8–244.1) Stage II/III (Activated): 4.3 cells/mm^2^ (IQR: 1.9–9.5) Stage III (Macrophage-like): 1.1 cells/mm^2^ (IQR: 0.4–2.4) Cortical regions had higher α-tocopherol levels than subcortical regions. Microglia activation suppression is significant in cortical regions, not in subcortical regions
Morris et al. (2015) [9]	α-tocopherol, γ-tocopherol	**Brain tocopherol levels and AD neuropathology (amyloid plaque and NFT)** Brain region Inferior Temporal Cortex Midfrontal Cortex Posterior Putamen Ventromedial Caudate Nucleus Across these regions: ↑ γ-tocopherol levels, ↓ Alzheimer’s pathology (plaques and NFT) α-tocopherol alone did not provide significant protection.
Dorey et al. (2023) [48]	α-tocopherol, δ-tocopherol, γ-tocopherol	**Regional Brain Tocopherol Levels in Alzheimer’s Disease:** AD: ↓ tocopherol level in grey and white matter (white matter greater deficiency)
Chan et al. (2024) [19]	α-tocopherol, γ-tocopherol	**Tocopherol Distribution of Centenarians** Centenarians: Temporal cortex—↑ Total α-tocopherol concentration RRR-αT was the dominant stereoisomer in all subjects (>50% of total α-tocopherol). Brain γ-tocopherol was much lower than α-tocopherol (not significantly correlated with plaques/tangles).
Paganini-Hill et al. (2023) [10]	Vitamin E	**Alzheimer’s Disease Brain Neuropathology** Brain weight None/Low ADNC Group (*n* = 70): 1138 ± 125 g Intermediate/High ADNC Group (*n* = 180): 1117 ± 123 g *p*-value: 0.23 (Not significant) Low, high and any intake of vitamin E were associated with a significantly reduced risk of developing Alzheimer’s disease brain pathology (amyloid plaques, NFT, neuritic plaques)
Livny et al. (2020) [54]	Vitamin E, α-tocopherol, β-tocopherol, δ-tocopherol, γ-tocopherol	**Vitamin E Intake vs. Brain Volume and Haptoglobin Genotype** Inferior Frontal Gyrus Volume: Hp 1-1 carriers: significant decrease in volume with higher Vitamin E intake. Hp 2-1 carriers: significant increase in volume with higher Vitamin E intake. Hp 2-2 carriers: no significant changes. Other Brain Regions: (Middle and Superior Frontal Gyri, Middle Temporal Gyrus) No significant changes in any Hp genotype. White Matter Hyperintensities (WMH): Hp 1-1 carriers: higher WMH volume, indicating greater white matter damage. Hp 2-2 carriers: lower WMH volume, suggesting better white matter integrity. Vitamin E Interaction with Hp Genotype: A significant interaction was observed between Vitamin E intake and Hp genotype on brain volume (*p* = 0.011). This suggests genotype-specific effects of Vitamin E on brain structure
Mehvari et al. (2016) [4]	Vitamin E	**Vitamin E Decreases Seizure Frequency and Interictal Sharp Waves in Epilepsy** 50% of patients in the Vitamin E group showed a decrease in positive EEG findings (reduction in interictal epileptiform sharp waves). In contrast, only 12.1% of patients in the placebo group showed similar improvements. Before Treatment: Both groups had a median of 2 seizures per month. After 6 Months: Vitamin E Group: Seizure frequency decreased to 1 seizure per month (*p* < 0.0001). Control Group: Seizure frequency remained at 2–3 seizures per month (*p* = 0.008).
Gopalan et al. (2014) [3]	Tocotrienol	**Tocotrienol supplementation significantly reduced the progression of WMLs over two years**. Placebo group: WMLs increased by 23.3% after 2 years. Tocotrienol group: WML volume remained unchanged (negligible change of −1.9%). Tocotrienol supplementation protected against WML progression, suggesting structural brain protection.

Abbreviation: α-, β-, γ-, δ- are isomeric forms of tocopherols and tocotrienols. RRR-αT = RRR-α-Tocopherol (natural stereoisomer); all-rac-α-tocopherol = Synthetic α-Tocopherol; HPC = Hippocampus; FC = Frontal Cortex; VC = Visual Cortex; TC = Temporal Cortex; PC = Parietal Cortex; NFT = Neurofibrillary Tangle; NP = Neuritic Plaque; AD = Alzheimer’s Disease; ADNC = Alzheimer’s Disease Neuropathologic Change; WMHs = White Matter Hyperintensities; Hp = Haptoglobin; SNARE = Soluble N-ethylmaleimide-sensitive factor Attachment protein Receptor; EEG = Electroencephalogram; WMLs = White Matter Lesions; IQR = Interquartile Range; BA = Brodmann Area.

**Table 4:** Summary of cross-sectional and observational studies examining the relationship between vitamin E forms (tocopherols and tocotrienols) and cognitive outcomes. This table includes the study source and year, type of vitamin E assessed, assessment method (e.g., blood, survey, brain tissue), and key cognitive-related findings.

Source (Year)	Vitamin E Form	Vitamin E Assessment Method	Cognitive Effect Outcomes
Chan et al. (2024) [19]	α-tocopherol, γ-tocopherol	Survey	◀▶ α-tocopherol levels between subjects with and without dementia. ▼: No direct association between α-tocopherol stereoisomers and premortem cognitive function
Boccardi et al. (2023) [13]	α-tocopherol, β-tocopherol, δ-tocopherol, γ-tocopherol, α-Tocotrienol, β-tocotrienol, δ-tocotrienol, γ-tocotrienol	Blood	Higher plasma α-Tocopherol level: ▲ Cognitive ability, daily living skills, instrumental tasks ▲ miR-122 expression ▲ inflammatory cytokines.
Alghadir et al. (2021) [51]	α-tocopherol, γ-tocopherol	Blood	▲ cognitive capacity has higher α-tocopherol and γ-tocopherol levels ◀▶ cognitive function in higher vitamin E.
Casati et al. (2020) [55]	Vitamin E, Tocopherol, α-tocopherol, β-tocopherol, δ-tocopherol, γ-tocopherol, Tocotrienol, α-Tocotrienol, β-tocotrienol, δ-tocotrienol, γ-tocotrienols	Blood	AD patients: ▼ lower plasma Vitamin E levels (α-, β-, γ-, δ-tocopherol and tocotrienols). ▼ poorer cognitive function ▼ Higher oxidative/nitrosative damage markers higher nitrosative stress (5-nitro-γ-tocopherol/γ-tocopherol ratio), linked to cognitive decline
Tanprasertsuk et al. (2018) [57]	α-tocopherol, γ-tocopherol	Blood; brain	▲ cognitive performance in higher Serum α-Tocopherol (Vitamin E) group ◀▶ cognitive performance in Serum γ-Tocopherol
Kim et al. (2018) [16]	Vitamin E, α-tocopherol, γ-tocopherol	Blood	▲ cognitive effect in Beta-Gamma Tocopherol (Middle Tertile) ▲ cognitive effect in Beta-Gamma Tocopherol in men ▲ cognitive effect in Beta-Gamma Tocopherol in non-drinkers ▲ cognitive effect in Beta-Gamma Tocopherol in non-smokers ◀▶ cognitive performance in Vitamin A, Vitamin C and α—tocopherol.
Huang et al. (2018) [58]	α-tocopherol, γ-tocopherol	Blood; survey	▼ poorer memory, executive function, and overall cognition. MCI subjects had higher α-tocopherol levels (MoCA scores)
Kuchan et al. (2016) [20]	α-tocopherol	Brain	Level of vitamin E in infant brain
Tanprasertsuk et al. (2021) [12]	α-tocopherol, γ-tocopherol	Brain	▲ Lower NFT count in early-affected brain—higher α-Tocopherol, ▲ Braak I-II in higher α-Tocopherol, ◀▶ NFT Counts in Late-Affected Brain Regions of α-Tocopherol ◀▶ α-Tocopherol vs. MMSE Scores ◀▶ γ-Tocopherol vs. NFT Counts ◀▶ γ-Tocopherol vs. MMSE Scores

Abbreviations: α-, β-, γ-, δ- are isomeric forms of tocopherols and tocotrienols. RRR-α-tocopherol = natural stereoisomer of α-tocopherol. PUFA = Polyunsaturated Fatty Acid; APOEɛ4 = Apolipoprotein E epsilon 4 allele; MIS = Memory Impairment Screen; TICS-m = Modified Telephone Interview for Cognitive Status; CERAD = Consortium to Establish a Registry for Alzheimer’s Disease; MoCA = Montreal Cognitive Assessment; MMSE = Mini-Mental State Examination; CDT = Clock Drawing Test; BTA = Brief Test of Attention; Aβ = Amyloid beta; E2 = Estradiol; AD = Alzheimer’s Disease; CIND = Cognitive Impairment, Not Dementia; Hp = Haptoglobin genotype. ▲ = Increase or positive association; ▼ = decrease or negative association; ◀▶ = no significant association or neutral effect. miR = microRNA.

**Table 5:** Summary of intervention and observational studies examining the association between Vitamin E intake (supplementation or dietary sources) and their effects on cognitive performance. The table includes the source and year of publication, form of vitamin E examined (e.g., α-, β-, γ-, δ-tocopherol or tocotrienol), the method of assessment (administered vs. dietary survey), dosage information (when available), and reported cognitive outcomes. Arrows represent the direction of observed associations: ▲ (positive improvement or protective effect), ▼ (negative or adverse effect), and ◀▶ (no significant change).

Source (Year)	Vitamin E Form	Vitamin E Assessment Method	Doses of Vitamin E Supplementation	Cognitive Effect Outcomes
Power et al. (2022) [50]	α-tocopherol (RRR-α-tocopherol) [as a part of mixture of intervention]	Administered	Co-supplementation: RRR-α-Tocopherol: 15 mg/day	◀▶ global cognition, episodic memory. ▲ working memory, executive function (attention, processing speed), language skills
Stavrinou et al. (2020) [53]	Vitamin E, γ-tocopherol	Administered	Co-supplementation: α-Tocopherol (Vitamin E): 22 mg/day γ-Tocopherol: 760 mg/day	Post supplementation: ▲ global cognition, memory performance ▲ executive function and processing speed ◀▶ cognitive flexibility ▲ language and fluency ▲ visuospatial abilities.
Sekikawa et al. (2020) [38]	Tocotrienol	Administered	Co-supplementation: Tocotrienols: 50 mg/day	▲ composite memory, verbal memory ◀▶ visual memory, executive function, processing speed, reaction time, cognitive flexibility, complex attention, social acuity, working memory, sustained attention, motor speed ▲ reduction in memory complaints
Bentsen et al. (2017) [59]	Vitamin E	Administered	Co-supplementation: RRR-α-tocopherol: 364 mg/day	▼ in high PUFA patients ◀▶ working memory
Kryscio et al. (2017) [18]	Vitamin E	Administered	Co-supplementation: Vitamin E (α-tocopherol): 400 IU/day	◀▶ dementia incidence ◀▶ memory function (MIS, TICS-m, CERAD) ◀▶ executive function ◀▶ learning and processing speed ◀▶ attention and decision-making ◀▶ overall cognitive decline prevention
Sano et al. (2016) [61]	Vitamin E	Administered	Vitamin E: 2000 IU/day	◀▶ in rate of decline, placebo vs. treatment ◀▶ in recognition, recall, memory and cognitive function, vocabulary test, behavior, function, and orientation ◀▶ dementia progression and cognitive decline ◀▶ global cognitive function
Remington et al. (2015) [64]	α-tocopherol	Administered	Co-supplementation α-tocopherol: 30 IU	▲ overall cognitive function ◀▶ executive function ▲ reduce the risk of cognitive decline
Remington et al. (2015) [65]	α-tocopherol	Administered	Co-supplementation α-tocopherol: 30 IU	▲ executive function, memory, and cognition, behavioral and mood, stabilized cognitive function ▲ improvement in delayed-start treatment
Nolan et al. (2022) [2]	α-tocopherol (RRR-α-tocopherol) [as a part of mixture of intervention]	Administered	Co-supplementation α-tocopherol: 15 mg	▲ slower cognitive decline, improvement in cognitive category, less decline in dementia severity, ▲ higher stability in dementia progression ▲ caregiver-reported memory: significant memory preservation
Li et al. (2015) [8]	α-tocopherol	Administered	Co-supplementation: Vitamin E (VE): 200 mg/day	◀▶ general cognitive function (memory, attention, language, orientation, and problem-solving ability) ◀▶ memory, calculation, and language comprehension. ◀▶ no reduction in plasma Aβ Levels and E2 levels
Dysken et al. (2014) [68]	α-tocopherol	Administered	Co-supplementation: Vitamin E: 2000 IU/day	◀▶ global cognitive function, including memory, attention, and orientation. (slightly slow decline, but no clinical difference) ▲ cognitive functions such as memory, language, and praxis ◀▶ after adjustment no statistically significant difference)
Zhang et al. (2023) [45]	α-tocopherol,	Survey	Average intake: Vitamin E: 8.1 mg/day	Higher α-Tocopherol Intake: ▲ cognitive performance: memory performance, verbal fluency, semantic memory, processing speed, attention, working memory
Liu et al. (2023) [46]	α-tocopherol, β-tocopherol, δ-tocopherol, γ-tocopherol	Survey	Total Vitamin E (from food only) Range (Q1–Q5 quintiles): Lowest quintile (Q1): ~4.15 mg/day Highest quintile (Q5): ~7.78 mg/day Maximum observed intake (from outliers): up to 43.45 mg/day α-Tocopherol (main form studied) Range: Q1: 5.52 mg/day Q5: 9.12 mg/day Maximum observed: up to 45.2 mg/day Other tocopherols: β-Tocopherol: up to ~2.91 mg/day γ-Tocopherol: up to ~30.2 mg/day δ-Tocopherol: up to ~9.76 mg/day	▲ global cognitive function, ▲ episodic memory, ▲ perceptual speed, ▲ cognitive performance, ▲ α-tocopherol protective effect in cognitive performance of APOEɛ4 Carriers. ◀▶ δ-tocopherol protective effect in cognitive performance of APOEɛ4 carriers.
Li et al. (2023) [47]	Vitamin E	Survey	Quartile Vitamin E Intake Range (mg/day) Q1: 0.06–2.188 mg/day Q2: 2.188–3.47 mg/day Q3: 3.47–5.442 mg/day Q4: 5.442–57.475 mg/day	▲ memory (short-term and long-term recall), executive function and verbal fluency, processing speed and verbal fluency, processing speed and attention, overall cognitive function in the higher vitamin E intake group ▲ cognitive performance on the higher vitamin E intake group
Livny et al. (2020) [54]	Vitamin E, α-tocopherol, β-tocopherol, δ-tocopherol, γ-tocopherol	Survey	Reported Intake Levels (from diet, not supplements): Hp 1-1: 12.62 mg/day (mean) Hp 2-1: 14.77 mg/day (mean) Hp 2-2: 15.05 mg/day (mean)	◀▶ on cognitive status for each Hp ▼ brain volume reduction for Hp 2-1
Beydoun et al. (2020) [56]	Vitamin E	Survey	Mean intake across all participants: 6.48 ± 4.62 mg/day of α-tocopherol (from food only). By carotenoid intake tertiles: T1 (lowest carotenoid intake): 4.64 ± 3.32 mg/day T2 (medium carotenoid intake): 6.39 ± 4.26 mg/day T3 (highest carotenoid intake): 8.46 ± 5.30 mg/day	▲ verbal memory in high carotenoid intake patients ◀▶ verbal memory in low carotenoid intake patients ▲ slowing down cognitive decline ◀▶ on CDT (executive function test) ▲ working memory in high carotenoid intake patients ◀▶ brief test of attention (BTA) ◀▶ psychomotor speed
Basambombo et al. (2017) [60]	Vitamin E	Survey	Vitamin E intake was self-reported, but no information on the dose.	▲ reduced risk of dementia ▲ reduced risk of AD ▲ better cognitive performance over time ▲ slower rate of cognitive decline ◀▶ cognitive impairment, not dementia (CIND)
Zhang et al. (2023) [49]	Vitamin E	Survey	Intake was analyzed by quartiles: Q1 (Lowest): ≤4.925 mg/day Q2: 4.925 to ≤7.25 mg/day Q3: 7.25 to ≤10.435 mg/day Q4 (Highest): >10.435 mg/day	The higher intake of Vitamin E group has ▲ memory (short-term and long-term recall), ▲ verbal fluency and executive function, ▲ processing speed and function, ▲ overall cognitive performance
Paganini-Hill et al. (2023) [10]	Vitamin E	Survey; Brain autopsies	Vitamin E intake was self-reported, but no information on the dose.	▲ lower odds of developing high AD neuropathology ▲ less likely to experience cognitive impairment and dementia ◀▶ similar benefits between low and high doses
Liu et al. (2023) [46]	Vitamin E	Survey	Total Vitamin E (from food only) Range (Q1–Q5 quintiles): Lowest quintile (Q1): ~4.15 mg/day Highest quintile (Q5): ~7.78 mg/day Maximum observed intake (from outliers): up to 43.45 mg/day α-Tocopherol (main form studied) Range: Q1: 5.52 mg/day Q5: 9.12 mg/day Maximum observed: up to 45.2 mg/day Other tocopherols: β-Tocopherol: up to ~2.91 mg/day γ-Tocopherol: up to ~30.2 mg/day δ-Tocopherol: up to ~9.76 mg/day	Higher vitamin E intake ▲ slower cognitive decline ▲ reduce dementia risk, in APOEε4 carriers too ▲ cognitive engagement (better brain health) ◀▶ depressive symptom

Abbreviations: α-, β-, γ-, δ- are isomeric forms of tocopherols and tocotrienols. RRR-α-tocopherol = naturally occurring stereoisomer of α-tocopherol. APOEɛ4 = Apolipoprotein E epsilon 4 allele; PUFA = Polyunsaturated Fatty Acid; Aβ = Amyloid beta; E2 = Estradiol; MIS = Memory Impairment Screen; TICS-m = Modified Telephone Interview for Cognitive Status; CERAD = Consortium to Establish a Registry for Alzheimer’s Disease; MoCA = Montreal Cognitive Assessment; MMSE = Mini-Mental State Examination; CDT = Clock Drawing Test; BTA = Brief Test of Attention; AD = Alzheimer’s Disease; CIND = Cognitive Impairment, Not Dementia; Hp = Haptoglobin genotype.

**Table 6:** Summary of disease-related outcomes associated with vitamin E forms (tocopherols and tocotrienols), as reported in human studies. The table outlines key biomarkers and functional indicators such as cognitive performance, amyloid plaques, tau pathology, APOE4 genotype, miRNA expression, presynaptic protein levels, neuroprotective effects, and miscellaneous outcomes on Alzheimer’s disease. Various neurological diseases include dementia, Parkinson’s disease, schizophrenia, psychosis, and epilepsy.

Diseases	Indicators	*Outcomes*
Alzheimer’s Disease	Cognitive performance	Refer to cognitive performance subtopics (Table 4 and Table 5)
	Amyloid plaques	[RRR-αT] higher amount correlated with fewer plaques in the frontal cortex and temporal cortex [19] [RSS-αT] higher amount correlated with more plaques in the temporal cortex [19] [γ-tocopherol] lowers amyloid plaque burden [1,9] When [γ-tocopherol] levels were low, higher [α-tocopherol] levels were linked to increased amyloid plaque burden [9] [α-Tocopherol] levels were not independently associated with Braak staging [9] No significant association between [α-tocopherol] levels and neuritic plaque (NP) counts in any brain region [12] Reduced AD neuropathology risk [10]
	TAU pathology (Neurofibrillary tangles)	[RSS-αT] higher amount correlated with increased NFTs in the frontal cortex, temporal cortex and amygdala [19] [RRR-αT] no significant correlation was found with NFTs. [γ-tocopherol] higher was associated with lower NFT severity [1,9] Higher [α-tocopherol] levels were associated with lower NFT counts in brain regions affected in early Braak stages (amygdala, hippocampus, entorhinal cortex, subiculum) [12] No significant association between [α-tocopherol] and [Vitamin E] levels in later-affected brain regions (frontal, temporal, parietal cortex) [10,12]
	APOE4	Genotype distribution in centenarians: ε3/ε3 (68%), ε2/ε3 (15%), ε3/ε4 (15%), ε2/ε4 (2%) [19] APOEɛ4 carriers with high dietary [α-tocopherol] intake had significantly slower cognitive decline [46] The protective effect of [tocopherols] and [vitamin E] remained even after adjusting for APOE4 status, suggesting it acts independently of genetic risk [1,10] No significant interaction was found between APOE4 and [tocopherol] levels in predicting amyloid or tangle pathology [9] APOE4 carrier status was recorded as a covariate, but it was not found to modify the relationship between [Vitamin E] levels and NFT counts [12]
	miRNA	[α-tocopherol] lower concentration is associated with lower miRNA expression. miR-122, miR-9, miR-21, miR-29b, miR-132 were significantly downregulated in AD patients [13]
	Presynaptic protein	Higher brain [γ-tocopherol] levels relate to higher levels of presynaptic proteins (SNARE protein composite, Complexin I, Complexin II, Syntaxin/SNAP-25 composite, Synaptotagmin, synaptophysin composite and Septin 5) in the midfrontal cortex [7]
	Neuroprotective	[RRR-αT] reduced plaque burden [19] [Vitamin E] slowed cognitive decline, particularly in APOEɛ4 carriers [46] [α-Tocopherol] lowered neuroinflammation [13] [tocopherol] lower activated microglia density provided an anti-inflammatory environment [1] AD subjects had lower [tocopherol and tocotrienol] levels and higher oxidative/nitrosative damage than CTs. Adjusted analyses confirmed these associations, with nitrosative damage linked to AD only in those with higher LTL [55] [γ-tocopherol] as a potential neuroprotective agent against Alzheimer’s disease pathology, while [α-tocopherol] alone is not effective [9] [α-tocopherol] may reduce early tau pathology but does not affect amyloid plaques [12]
	Misc.	managing sleep apnea might help prevent cognitive decline, particularly in APOE ɛ4 noncarriers
	Total homocysteine (tHcy)	No direct correlation between vitamin E and tHcy. However, higher physical activity was associated with lower tHcy levels [51]
Dementia	[Vitamin E] did not slow the progression of dementia or cognitive decline in aging individuals with Down syndrome [61] Lower [Vitamin E] intake (<13.20 mg/day) was associated with a significantly higher incidence of dementia. Higher dietary [Vitamin E] intake (>23.63 mg/day) was associated with a lower risk of dementia and a slower cognitive decline [29] [γ-tocopherol] association with synaptic integrity remained significant even in individuals with infarcts, indicating potential benefits for vascular dementia [7] Did not affect the relationship between [γ-tocopherol] and presynaptic protein levels, implying that vitamin E may protect synapses even in individuals with Lewy body-related dementia [7]	
Parkinson’s Disease	Higher dietary [Vitamin E] intake may be associated with a lower risk of developing Parkinson’s disease [44], without influencing disease severity [11] Older age and comorbidities (hypertension, CVD) increase PD risk. No significant associations were found with smoking, alcohol, BMI, marital status, or education [44] Neuroaspis PLP10™ significantly delayed Parkinson’s disease motor symptom progression and reduced the need for increased levodopa doses [52].	
Schizophrenia	Combining EPA and [Vitamin E] +C lessen psychotic symptoms and/or disturbed sustained attention [59]	
Psychosis	PUFA-E supplementation led to antioxidant changes (higher α-tocopherol, altered glutathione levels) but did not significantly impact PANSS scores or psychotic symptoms within 12 weeks [63]	
Seizure and Epilepsy	Seizure frequency significantly decreased in the [Vitamin E] group compared to the placebo group. EEG findings improved significantly, suggesting better neuronal stability [4]	

Abbreviation: AD = Alzheimer’s Disease; APOEɛ4 = Apolipoprotein E epsilon 4 allele; α-, β-, γ-, δ- = isomeric forms of tocopherols and tocotrienols; RRR-αT, RSS-αT = natural and synthetic stereoisomers of α-tocopherol; NP = Neuritic Plaque; NFT = Neurofibrillary Tangle; miR = microRNA; SNARE = Soluble NSF Attachment Protein Receptor complex; CTs = Control Subjects; LTL = Leukocyte Telomere Length; tHcy = Total Homocysteine; EEG = Electroencephalogram; PANSS = Positive and Negative Syndrome Scale; PUFA = Polyunsaturated Fatty Acid; EPA = Eicosapentaenoic Acid; CVD = Cardiovascular Disease; BMI = Body Mass Index.

## Data Availability

The data presented in this study is available on request from the corresponding author.

## Abbreviations

The following abbreviations are used in this manuscript:

AD	Alzheimer’s Disease
ADCS-ADL	Alzheimer’s Disease Cooperative Study Activities of Daily Living
ADL	Activities of Daily Living
AE/SAE	Adverse Event/Serious Adverse Event
AF/AFT	Animal Fluency Test
APOE	Apolipoprotein E
BDNF	Brain-Derived Neurotrophic Factor
BPSD	Behavioral and Psychological Symptoms of Dementia
BVRT	Benton Visual Retention Test
CAS	Caregiver Activity Survey
CDR	Clinical Dementia Rating
CERAD	Consortium to Establish a Registry for Alzheimer’s Disease
CES-D	Center for Epidemiologic Studies Depression Scale
CLOX-1	Clock Drawing Executive Function Test
CRP	C-reactive protein
DHA	Docosahexaenoic Acid
DRS	Dementia Rating Scale
DS-B	Digit Span Backward
DS-F	Digit Span Forward
DSRS	Dementia Severity Rating Scale
DSST	Digit Symbol Substitution Test
FORD	Free Oxygen Radical Defense
FORT	Free Oxygen Radical Test
GAF	Global Assessment of Functioning
GDS	Geriatric Depression Scale
GM-CSF	Granulocyte-Macrophage Colony-Stimulating Factor
GSHt/GSHr/GSSG	Total, Reduced, and Oxidized Glutathione
Hp	Haptoglobin
ICD	International Classification of Diseases
IL	Interleukin
INF-α2/IFN-γ	Interferon-alpha2/Interferon-gamma
LOTCA	Loewenstein Occupational Therapy Cognitive Assessment
LTL	Leukocyte Telomere Length
MCI	Mild Cognitive Impairment
MMSE	Mini-Mental State Examination
MoCA	Montreal Cognitive Assessment
NFT	Neurofibrillary Tangles
NPI	Neuropsychiatric Inventory
PANSS	Positive and Negative Syndrome Scale
PASAT	Paced Auditory Serial Addition Test
PHQ-9	Patient Health Questionnaire-9
PRISMA-ScR	Preferred Reporting Items for Systematic reviews and Meta-Analyses Scoping Review
PUFA	Polyunsaturated Fatty Acid
RBANS	Repeatable Battery for the Assessment of Neuropsychological Status
RNS/ROS	Reactive Nitrogen Species/Reactive Oxygen Species
SNARE	Soluble N-ethylmaleimide-sensitive factor Attachment protein Receptor
SOD	Superoxide Dismutase
STROOP	Stroop Color and Word Test
TAC	Total Antioxidant Capacity
TICS-m	Modified Telephone Interview for Cognitive Status
TMT	Trail Making Test
TNF-α/TNF-β	Tumor Necrosis Factor alpha/beta
UPDRS III	Unified Parkinson’s Disease Rating Scale Part III
VEGF	Vascular Endothelial Growth Factor
VLDL	Very Low-Density Lipoprotein
WMHs	White Matter Hyperintensities
WMLs	White Matter Lesions
